# RNA sequencing of transcriptomes in human brain regions: protein-coding and non-coding RNAs, isoforms and alleles

**DOI:** 10.1186/s12864-015-2207-8

**Published:** 2015-11-23

**Authors:** Amy Webb, Audrey C. Papp, Amanda Curtis, Leslie C. Newman, Maciej Pietrzak, Michal Seweryn, Samuel K. Handelman, Grzegorz A. Rempala, Daqing Wang, Erica Graziosa, Rachel F. Tyndale, Caryn Lerman, John R. Kelsoe, Deborah C. Mash, Wolfgang Sadee

**Affiliations:** Center for Pharmacogenomics, College of Medicine, The Ohio State University, Columbus, OH 43210 USA; Department of Biomedical Informatics, College of Medicine, The Ohio State University, Columbus, OH 43210 USA; Division of Biostatistics, College of Public Health, and Mathematical Biosciences Institute, The Ohio State University, Columbus, OH USA; Thermo Fisher Scientific, South San Francisco, CA 94080 USA; Center for Addiction and Mental Health and Departments of Psychiatry and Pharmacology & Toxicology, University of Toronto, Toronto, Ontario Canada; Department of Psychiatry, Annenberg School for Communication, and Abramson Cancer Center, University of Pennsylvania, Philadelphia, PA USA; Department of Psychiatry, Laboratory of Psychiatric Genomics, University of California, San Diego, USA; VA San Diego Healthcare System, La Jolla, San Diego, CA USA; Department of Neurology, Miller School of Medicine, University of Miami, Miami, FL 33136 USA; Departments of Pharmacology, College of Medicine; Colleges of Pharmacy and Environmental Health Sciences, The Ohio State University, Columbus, OH USA; Departments of Psychiatry, College of Medicine; Colleges of Pharmacy and Environmental Health Sciences, The Ohio State University, Columbus, OH USA; Departments of Human Genetics/Internal Medicine, College of Medicine; Colleges of Pharmacy and Environmental Health Sciences, The Ohio State University, 5078 Graves Hall, 333 W. 10th Avenue, Columbus, OH 43210 USA

**Keywords:** RNA sequencing, Brain regions, Differential expression, Allelic expression imbalance, Isoform fraction, Non-coding RNA

## Abstract

**Background:**

We used RNA sequencing to analyze transcript profiles of ten autopsy brain regions from ten subjects. RNA sequencing techniques were designed to detect both coding and non-coding RNA, splice isoform composition, and allelic expression. Brain regions were selected from five subjects with a documented history of smoking and five non-smokers. Paired-end RNA sequencing was performed on SOLiD instruments to a depth of >40 million reads, using linearly amplified, ribosomally depleted RNA. Sequencing libraries were prepared with both poly-*dT* and random hexamer primers to detect all RNA classes, including long non-coding (lncRNA), intronic and intergenic transcripts, and transcripts lacking poly-*A* tails, providing additional data not previously available. The study was designed to generate a database of the complete transcriptomes in brain region for gene network analyses and discovery of regulatory variants.

**Results:**

Of 20,318 protein coding and 18,080 lncRNA genes annotated from GENCODE and lncipedia, 12 thousand protein coding and 2 thousand lncRNA transcripts were detectable at a conservative threshold. Of the aligned reads, 52 % were exonic, 34 % intronic and 14 % intergenic. A majority of protein coding genes (65 %) was expressed in all regions, whereas ncRNAs displayed a more restricted distribution. Profiles of RNA isoforms varied across brain regions and subjects at multiple gene loci, with neurexin 3 (NRXN3) a prominent example. Allelic RNA ratios deviating from unity were identified in > 400 genes, detectable in both protein-coding and non-coding genes, indicating the presence of *cis*-acting regulatory variants. Mathematical modeling was used to identify RNAs stably expressed in all brain regions (serving as potential markers for normalizing expression levels), linked to basic cellular functions. An initial analysis of differential expression analysis between smokers and nonsmokers implicated a number of genes, several previously associated with nicotine exposure.

**Conclusions:**

RNA sequencing identifies distinct and consistent differences in gene expression between brain regions, with non-coding RNA displaying greater diversity between brain regions than mRNAs. Numerous RNAs exhibit robust allele selective expression, proving a means for discovery of *cis*-acting regulatory factors with potential clinical relevance.

**Electronic supplementary material:**

The online version of this article (doi:10.1186/s12864-015-2207-8) contains supplementary material, which is available to authorized users.

## Background

The architecture and connectivity of brain regions critically influence CNS functions, including cognition, behavior, decision making and emotional control. Deregulation of dynamic CNS processes lead to psychiatric disorders, including depression, schizophrenia, and addiction. A mirror of the dynamic biological processes underlying brain functions, RNA transcript profiles have been measured in numerous studies, mostly with focus on protein-coding mRNAs, using targeted analysis or cDNA hybridization technology [1–3]. However, less than 2 % of the human genome accounts for protein-coding transcripts, while a large portion of the genome expresses non-coding RNAs, implicated in multiple biological roles regulating gene expression, guiding epigenetic processes, sensing cellular substrates, serving as catalysts or enzymes, and supporting structural functions [4]. Moreover, a majority of disease risk alleles implicated by genome-wide association studies (GWAS) reside outside protein coding exons, affecting transcription of all RNA types and RNA processing—areas still incompletely resolved [5, 6].

Next generation sequencing of RNA profiles (RNAseq) has opened the door for systematic exploration of the entire transcriptome, including genetic and epigenetic factors, and regulatory networks that often cannot be a reconstructed from protein-coding RNAs alone [2, 7, 8]. While this technology is rapidly maturing, different technology platforms and tissue preparation procedures have strong effects on results and interpretation [7, 9]. Recent studies have explored the transcriptome of the human brain, with increasing use of RNAseq, comparing brain autopsy regions from subjects with no previous diagnosis of a CNS disorder with regions from subjects with various diagnoses such as schizophrenia, alcohol dependence, and chronic nicotine exposure [8, 10–15]. More detailed analyses have been done with laser micro-dissection to minimize issues arising with RNAseq data obtained from heterogeneous regions [15]. Where use of heterogeneous regions cannot be avoided, computational deconvolution of co-expression gene networks can serve to dissect expression profiles for cellular subtypes [16]. Together, studies on CNS transcriptome profiles have revealed a wealth of candidate genes implicated in CNS functions and disorders.

In this study, we have measured RNAseq profiles in 10 brain regions from 10 human subjects, to generate a database for regional expression and inter-individual variability. Moreover, our study provides detailed data on RNA expression profiles, reflecting all types of RNA classes and RNA isoforms at each gene locus, supplementing existing studies involving human brain regions using hybridization arrays and large-scale genotyping, revealing multiple *cis*-acting quantitative expression traits (*cis*-eQTLs) and SNPs associated with *CpG* methylation patterns [1, 3, 13, 17, 18]. With microarrays using probes for multiple exons per gene, *cis*-eQTLs were found to be frequently associated with only some exons in a given gene, implicating a pervasive genetic influence on splicing [3], which is often region specific [19, 20]. However, microarray analysis is limited in detecting RNA transcript isoforms, whereas deep sequencing reveals the rich abundance of isoforms at each gene locus [7, 21].

RNAseq is typically performed with pol*y-*dT to capture poly-adenylated RNA transcripts—these include most protein-coding mRNAs and numerous ncRNAs, but numerous RNAs do not carry a poly*-A* tail. To account for the emerging functions and interactions of all RNA classes, including non-coding RNAs, we have applied RNAseq in a process that captures all transcripts, regardless of polyadenylation status [7]. In this report, we focus on long RNAs (>200 bases), owing to the available RNAseq protocols that require a separate approach for measuring small RNAS, such as microRNAs—these will be reported in a subsequent study. Owing to the use of random hexamer primers in this study that captures non-polyadenylated RNAs as well, we were also interested in determining the relative abundances of the various RNA classes, protein-coding and non-coding, across brain regions.

Use of RNAseq enables us to measure transcript abundance and RNA isoforms, such as splice variants, different 3′ and 5′ UTRs, and edited RNAs [7, 21]. In addition, we have developed a quantitative approach to exploit RNAseq data for measuring allelic RNA expression ratios, a sensitive indicator of regulatory variants affecting gene expression and RNA processing [22]. To enable full analysis of allelic RNA expression, we have also applied whole-genome SNP chip analysis, as reported before in detecting *cis*-eQTLs [3, 6, 13], facilitating allele calling in RNA transcripts, detecting cases of RNA editing [22] and of mono-allelic expression, and alerting to the presence of copy number variants.

Of the ten subjects in this study, five were previous long-term smokers and 5 were non-smokers, all lacking other diagnoses of psychiatric disorders. While the number of subjects is small for detecting nicotine-related changes, we expected to develop leads for further study because nicotine exerts rather robust effects on cellular biology throughout the body [3, 23]. Our analyses were guided by previous studies on the influence of nicotine on brain transcriptomes, genetic effects on nicotine related genes [3, 23–25], and candidate genes associated with smoking from multiple studies [26]. By comparing RNA profiles in different human autopsy brain regions, we initiate here a comprehensive study of the brain transcriptome, interactive networks between RNAs, and genetic factors regulating expression, with nicotine exposure serving as a perturbing environmental stimulus.

## Methods

### Postmortem human brain tissues

Brain tissue samples from five male subjects with a history of heavy cigarette smoking and five age-matched male drug-free controls were provided by the Miami Brain Endowment Bank^TM^ (University of Miami, Miami, FL) following protocols approved by the research ethics board of the University of Miami Miller School of Medicine [27–32]. Brain biospecimens were banked from persons at autopsy. The authorization for retention of brain and tissues, medical records review, and informant interviews were approved by the University of Miami Institutional Review Board (Protocol No. 19920580). Ethical procedures including donor anonymity are assured. All brain tissue is procured, stored, and distributed according to applicable regulations and guidelines involving consent, protection of human subjects and donor anonymity. The genomics analyses on the de-identified autopsy brain tissues were exempt from IRB approval at OSU. Supplemental brain and blood toxicology and neuropathologic evaluations were done in every case. Subjects were selected from accidental or cardiac sudden deaths with negative urine screens for all common drugs, except nicotine, and there was no history of psychiatric or medical disorders or licit or illicit drug use prior to death. From each subject, ten brain regions were obtained to provide a diverse set of brain regions: frontopolar cortex (Brodmann Area 10; BA10), Wernicke’s area (BA22), anterior cingulate cortex (BA24), dorsolateral prefrontal cortex (BA46), insular cortex, hippocampus, amygdala, posterior putamen, cerebellum, and brainstem raphe nuclei.

### RNA preparation

Frozen brain tissue was homogenized in Trizol (Invitrogen/Life Technologies, Carlsbad, CA), and then phase separated with chloroform. The RNA containing aqueous layer was diluted in binding buffer and applied to Qiagen (Venlo, Limburg, Netherlands) or Denville (South Plainfield, NJ) RNA isolation columns. The bound RNA was DNAse treated and eluted according to kit procedures. RNA concentration was measured using Qubit (Invitrogen/Life Technologies, Carlsbad, CA), and integrity assessed by Bioanalyzer (Agilent, Santa Clara, CA). Samples with RIN numbers >6 were used for analysis.

### cDNA synthesis

Fifty nanograms of total RNA was converted to cDNA and isothermally amplified using the NuGen Ovation RNA-Seq kit procedures (NuGen, San Carlos, CA). NuGEN’s proprietary SPIA technology is an elegant method for robust isothermal amplification of nucleic acids. Primer design strategies enable selective depletion of ribosomal RNA while amplifying all remaining coding and non-coding trancripts regardless of polyadenylation status. The resulting double stranded cDNA can be made into NGS libraries in a streamlined workflow bypassing any sequence enrichment procedure, leading to improved efficiency, throughput and data quality. Typically, 50 ng of input total RNA yielded 3–6 micrograms of double stranded cDNA. Ribosomal RNA was reduced to 3–5 % by the NuGen process. The yield of cDNA produced by isothermal amplification typically eliminates the need for additional PCR cycles, thus greatly reducing PCR duplicates in the final libraries.

### Library preparation

The NuGen brain cDNA was sheared to approximately 150 bp fragments using the Covaris S (Woburn, MA). After shearing, fragments were recovered by centrifuging over an YM-30 spin filter (Amicon, Merck Millipore, Billerica, MA). Fragments greater than 100 bp were retained and eluted from the membrane, with ~ 90 % recovery. Bar-coded paired-end SOLiD sequencing libraries were prepared using either SOLiD (Applied Biosystems/Life Technologies, Carlsbad, CA) or NEB (New England Biolab, Ipswich, MA) DNA library preparation kits. The cDNA was end repaired, and then barcoded SOLiD DNA sequencing adaptors were ligated to 1 microgram of input cDNA according to kit instructions. Ligated library product was size selected using a Pippin gel electrophoresis system (Sage Biosciences, Beverly, MA). The prepared library was enriched for correctly adapted product using 5–8 cycles of PCR. Library PCR product was analyzed for appropriate size distribution with the Bioanalyzer, and quantitated using qPCR with the library adaptors on an Applied Biosystems 7500 Real-Time instrument. To confirm RNAseq measured expression, qRTPCR based expression was calculated as ΔΔCt with three invariable genes (AGO1, SPEN, SRSF11) averaged for baseline normalization.

### Emulsion PCR, enrichment, slide preparation

To prepare libraries for sequencing on the SOLiD 4 or SOLiD 5500XL, the SOLiD EZ Beader System (Applied Biosystems/Life Technologies, Carlsbad, CA) was used for emulsion PCR and templated bead enrichment. Enriched beads were chemically bound to treated SOLiD flow cells, then sequenced using the SOLiD (sequencing by ligation) pairedend sequencing process. Some sets of RNA samples were prepared for sequencing on a SOLiD Wildfire instrument (Life Technologies, Carlsbad, CA), eliminating the need for emulsion PCR and library bead enrichment. Barcoded SOLiD libraries were prepared as before, and then Wildfire adapters were ligated to the SOLiD libraries. Calibrated concentrations of these Wildfire libraries were pipetted into the Wildfire flowchip. On-slide isothermal template walking produced defined, single insert colonies of appropriate size and density for SOLiD Wildfire paired end sequencing.

### Sequencing experimental design

Our goal was to sequence RNA from 10 brain regions in 10 subjects, at sufficient depth to detect a wide range of transcript expression, including non-coding RNA’s. The scope of the project required multiple sequencing runs. To account for sequencing batch effects, the sequencing runs were designed to optimize comparisons both between samples, and between regions. To facilitate these comparisons, nine of the brain regions from one subject were barcoded, combined, and sequenced together in the same run. The tenth brain region (BA46) from every subject was separately barcoded, and these barcoded samples from all ten subjects were sequenced together in one run. Of the 11 total runs, 8 were processed with Wildfire technology, and 2 runs were sequenced for the forward reads only. Several sets were replicated to account for changes in sequencing technology and to increase read coverage (Average Pearson correlation of libraries within a set across any replicates: MB52 = 0.70, MB160 = 0.94, MB147 = 0.98, MB100 = 0.85, MB11 = 0.86).

### Data processing

Sequence Alignments. RNAseq data were aligned to a modified version of the Genome Reference Consortium human genome build 37 (hg19, Feb. 2009) containing IUPAC ambiguous nucleotide characters for each annotated SNP in dbSNP 135 with alignment performed using SOLiD LifeScope™ Genomic Analysis Software v2.5.1 (Life Technologies Carlsbad, CA). Gene features were annotated using a combination of GENCODE v18 [33] annotation plus non-identical transcripts annotated by lncipedia v2.1 [34]. The combination of these annotations provides a richer set of coding and non-coding transcript types. The bedtools suite [35] was used to generate non-overlapping exonic, intronic, and intergenic annotation. The exonic regions for each transcript were merged and subtracted from the whole gene length to extract intronic regions. Whole genes were subtracted from the genome to identify intergenic regions. With these regional annotation sets, bedtools coverage was used to generate a count for each region and the proportion of coverage.

### Gene expression

Cufflinks v2.1.1 [36] was implemented to estimate gene specific abundances. Expression level is reported as FPKM (Fragments Per Kilobase per Million reads) which normalizes the number of reads within a gene by the number of fragments per kilobase of exon and million mapped reads for a given sample. Expression measurements were quantified strictly based on GENCODE v18 [33] gene annotation combined with the additional transcripts present only in the lncipedia non-coding RNA database [34]. The combination of these two annotation sets allowed for the consideration of a wide variety of protein coding and non-coding transcripts. Multi-read correction was applied to improve the expression estimates when considering multimapped reads. Whole gene expression measurements were based on the sum of the expression of all exons of all annotated isoforms at a gene locus.

### Entropy based analysis of stably expressed RNAs

Comparing results from multiple RNAseq runs requires means for normalization, accounting for batch effects and sample-to-sample variability. Stably expressed genes can serve to account for these confounding effects. Genes with stable expression across brain regions and subjects were identified as possible reference RNAs for normalizing expression levels of other RNAs using the following information-theoretic approach. First we searched for well-expressed genes (>3 FPKM) with a flat expression profile over the set of all available brain regions. Second, we have looked for stably expressed gene in each regions separately, taking the expression vector of each gene over the available individuals. For both genes with stable expression across all regions and genes with stable expression within a regions, we have treated every expression vector as a trial for a multinomial distribution, using Shannon entropy function as a measure of uniformity. For any probability distribution, entropy is always positive and attains its maximum (over the space of all discrete distributions with given support) on the uniform distribution [37]. Third, we have searched for well-expressed genes with reproducible expression patterns over the available individuals. We make our predictions more robust to inter-individual variability by using the following re-sampling-based procedure: we select m random subsets of k individuals (taking k = 2,3,…,9 and m between 10 and 50 dependent on the choice of k) and compared the selected k expression vectors (of a single gene over the available tissues). To compare expression vectors we have used the approach proposed by Rempala and Seweryn [38] and quantified the overlap between the k columns in a contingency table by calculating the I-index. The I-index is an overlap measure as a function of the mutual information; it is always positive and attains its maximum (which equals 1) if any of the columns in a contingency table are linearly dependent. We have selected the genes with reproducible expression patterns by comparing the minimum observed I-index over all selected sub-samples.

### Compilation of nicotine related genes

Genes related to nicotine exposure and addiction were gathered from several online sources. 1196 genes with expression differences related to smoking, nicotine, and tobacco were selected from the Expression Atlas (ebi.ac.uk) [39]; 60 genes with SNPs associated with smoking, smoking cessation, and tobacco use disorder were identified from GWAS and NCBI databases by PheGenI (ncbi.nlm.nih.gov/gap/phegeni) [40]; 40 genes with connections to nicotine and tobacco use were listed in PharmGKB [41]. Liu et al. proposed a list of 587 genes relating to nicotine, with a prioritized set of 220 genes [26]. A study by Tyndale et al. identified 58 genes relating to smoking cessation [42]. Of these a total of 1789 unique genes were mapped to GENCODE gene annotation.

### Differential RNA expression and interpretation

The read count per gene for analysis was generated by *featureCounts* from the subread package [43]. The primary alignment for each read was used in counting. Differential expression analysis was performed by edgeR [44] and RUVseq [45]. RUVseq used internally identified 200 ‘invariable’ genes to reduce variability between samples and estimated a term for edgeR’s glm analysis. Differential expression was performed pairwise between regions and between smokers and nonsmokers within a region. To be included in analysis between regions, a gene needed >10 reads in >8 samples. To be included in analysis between smokers and nonsmokers, we required a gene to have an expression of ≥ 2 counts per million (reads per gene divided by million aligned reads) in all subjects included in a comparison. GO term enrichment was performed with the ToppFun application of the ToppGene [46] suite to identify molecular and biological processes over-represented in the gene list. Custom pathways were built with Ingenuity Pathway Analysis (IPA®, QIAGEN Redwood City, www.qiagen.com/ingenuity) to find connections between RNA molecules and smoking/nicotine.

### SNP calling and allele specific expression

Genotyping was performed on Illumina GeneChip on genomic DNA for each of the 10 subjects. To overcome a bias in alignment of short reads, where the reference allele reads are preferentially aligned over reads with the variant allele, we used a genomic reference containing IUPAC codes for SNPs in dbSNP. This approach limits consideration to known SNPs, but equalizes the alignment rate of reads containing known variants. Default settings of samtools mpileup [47] were applied to each RNA library individually to make SNP calls only for heterozygous SNP locations identified by GeneChip. Gene bins were created for all annotated genes from the combined GENCODE and lncipedia annotation, taken as 1 Kb upstream and 1 Kb downstream of each annotated gene (recognizing that regulatory variants can be much more distant). Overlapping genes containing exactly the same SNPs will have the same AEI fold change value. For the analysis of allelic mRNA expression differences, SNPs were assigned to bins and could belong to multiple bins in the case of overlapping regions. A set of filters was applied to reduce the number of false positives arising from noise of the RNAseq data, guided by earlier quantitative estimates [24]. We retained SNPs belonging to at least one bin and having an assigned rs number based on dbSNP build 135, and filtered for a combined read coverage of 10 reads (reference allele count plus variant allele count). For the second level of filtering, we require a SNP to be called in 3 or more regions of the same subjects. Out of these, we selected genes that had two or more SNPs called within a sample from a tissue, to obtain at least two independent allelic ratio measurements for any given RNA. To meet this condition, we required the distance between SNPs in a gene to be greater than the length of a single read (>50 bases). For each gene, we calculated the average allelic ratio and read depth of coverage. RNAs with likely allelic expression imbalance greater than 2, regardless of individual SNP differences, were required to have an allelic ratio greater than twofold at the lower bound of the 95 % confidence interval. For a list of the highest confidence SNPs, we further filtered by an average read depth per SNP of 30 reads.

### RNA isoform analysis

Cufflinks v2.2.1 [36] was implemented to estimate isoform specific abundances using cuffquant to quantify expression, and cuffnorm to normalize the expression levels for RefSeq annotated isoforms downloaded from the UCSC genome browser in refFlat format. RefSeq was used for isoform quantitation because of its simplicity in transcript annotation compared to GENCODE. Isoform fraction is calculated as the expression of a single isoform divided by the sum of expression of isoforms assigned to a gene. Genes were considered when having ≥ 5 FPKM in at least 40 libraries. To detect genes generating different isoform patterns in different brain regions we sorted genes with multiple isoform by the number of tissues with an isoform average outside the 99 % confidence interval generated from all samples. This provided a simple measure of how much individual regions deviated from the average.

## Results

### Sequence read distribution across genomic regions

**Fig. 1:**
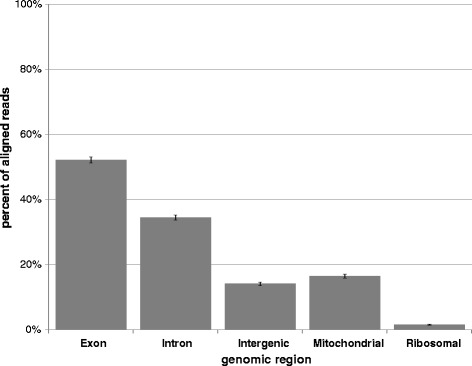
Read alignment across genomic regions. Presents the percentage of aligned reads falling within genomic regions of different types--exonic, intronic, and intergenic as annotated by GENCODE and lncipedia; any reads aligning to the mitochondrial chromosome; and ribosomal reads filtered by during alignment (18S, 28S, and 5.8S only)

Brain tissues were divided into 11 sequencing sets, with 10 sets including 9 regions for a single subject and 1 set including 9 subjects for one region. Replicate sets correlated well so the sequence reads were merged (Pearson correlation between replicate sets, reported as average across libraries within a set: MB52 = 0.70, MB160 = 0.94, MB147 = 0.98, MB100 = 0.85, MB11 = 0.86). Additional file 1: Table S1 provides complete sequencing and mapping information. After alignment with Lifescope, 60–80 % of forward reads and 40-70 % for reverse reads were aligned to genomic sequence. Separating aligned sequences by exonic, intronic, and intergenic regions yields estimates of read number generated from each region illustrated in Fig. 1. On average, 52, 34, and 14 % of reads aligned to exonic, intronic, and intergenic regions, respectively. These read counts are independent of the length of a gene locus or region. 15 % of reads aligned to the mitochondrial chromosome, and 2 % aligned to the three major ribosomal transcripts (18S, 28S, and 5.8S; reduced over 95 % with the NuGen kit). To account for length of genomic regions, we estimated RPKM (reads per kilobase per million total reads of a sample) per region by dividing total reads per genomic region by the total genomic length (kb) of each region annotated by GENCODE and lncipedia including both coding and non-coding RNAs, yielding an average expression of 4.2 RPKM, 0.2 RPKM, and 0.1 RPKM in exonic, intronic, and intergenic regions, respectively. By the same criteria, mitochondrial genes are highly expressed.

### Identification of genes with consistent expression across regions and samples

We first identified RNAs stably expressed across multiple tissues and subjects. These genes are useful as “normalizing” genes to facilitate comparison between samples. Mathematical modeling served to characterize global transcript expression patterns. Shannon entropy-based analysis was used to identify the RNAs that were similarly expressed across all brain regions in any individual, and those stably expressed in all regions in all 10 individuals (Additional file 1: Table S2). Additional file 2: Figure S1 shows the average expression of all invariable genes in the 4 GTEx brain regions that overlap the current survey confirming the expectation that these genes have a small range of expression across different brain regions. GO term analysis indicates that these “stable” and “invariable” genes are involved in various biological processes, such as, chromatin modification, (*BAZ1B*, *CHD2* and *MECP2*); mRNA processing (*SRRM1*, *RBM25*, *CPSF7*) and neuronal cell adhesion (*CDK5R1*, *NLGN2*, *ASTN1*). We propose that any of these genes could serve to normalize expression profiles, and used the genes to remove unwanted variation during differential expression analysis.

### Region-selective expression of RNA classes

**Fig. 2:**
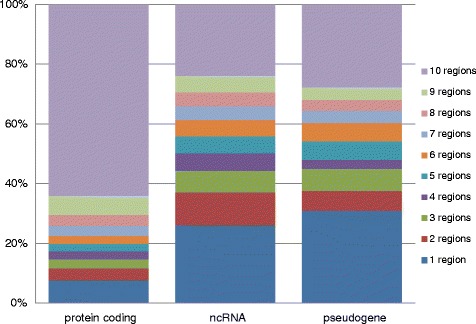
Brain region specificity of RNA classes. Presented is the percentage of different RNA types, as annotated in GENCODE/lncipedia, detectable across brain regions. Detectability is defined as FPKM > 2 in 2+ samples. This includes 10,680 protein coding genes, 242 pseudogenes, and 945 noncoding. A higher percentage of protein coding RNAs are detectable across all 10 regions compared to non-coding RNAs and pseudogenes

To test how RNA transcripts tend to be expressed across region types, we generated counts of the number of times an RNA is detectable across the 10 regions and stratified this by transcript-type based on GENCODE-lncipedia annotations. Detection within a region was defined as expression greater than 2 FPKM in two or more subjects. This arbitrary cutoff serves as an example to assess relative expression selectivity for various RNA classes, leaving out relevant transcripts with low expression. The relative contributions of reads aligned to protein coding, lncRNA, pseudogenes, and processed transcripts to regional expression patterns is shown in Fig. 2. The mRNAs represent by far the largest group of these relatively robustly expressed genes (10,680), followed by lncRNAs (838). lncRNAs include transcripts labeled as lincRNA, antisense, sense intronic, sense overlapping, and those added from lncipedia. Among the protein coding mRNAs, the majority is widely expressed, whereas other transcripts, in particular the lncRNAs, display a more selective expression pattern, possibly suggesting distinct functions of a relatively small set of non-coding RNAs required in various brain regions.

**Fig. 3:**
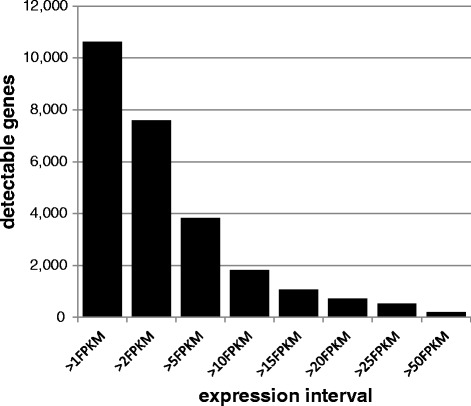
Number of detectable RNAs at different FPKM cutoffs. The average number of detectable protein coding and non-coding RNAs is shown at different expression cutoff levels. This illustrates the working pool of RNAs available depending of expression cutoff

To explore the number of detectable RNA transcript types across the brain, we calculated the average expression of each gene across all subjects. For further analysis, we created bins of expression levels ranging from 2 FPKM to 5000 FPKM (Fig. 3), leaving out RNAs with low expression levels (<2 FPKM). Read counts attributable to non-coding transcripts are generally lower than read counts aligned to protein coding transcripts. Half of expressed protein-coding RNAs are detectable with > 5 FPKM, whereas for lncRNA only 35 % of expressed RNAs are detectable at the 5 FPKM level. Hence, only a small number of ncRNAs displays robust expression, but these could be instrumental in defining specific functions across brain regions.

### Differential RNA expression across brain regions and subjects

RNAs with divergent expression patterns between brain regions and subjects likely reflect dynamic processes. To be included in analysis, we used RUVseq suggested filtering requiring >10 reads in >8 samples (at least half of all samples included in a comparison). FDR correction was made only for genes included in the analysis. In region to region comparisons, 8 to 10 subjects were included per category. We performed differential gene expression analysis with edgeR together with RUVseq in order to use invariant genes to reduce unwanted variation.

Table 1 shows the number of RNAs significantly (FDR ≤ 0.05) differentially expressed between every combination of region pairs. This analysis revealed relative similarities between regions - BA10, BA22, BA24, and insula (0–342 differentially expressed (DE) genes); amygdala and hippocampus (250 DE genes); and putamen, cerebellum, and raphae nucleus (0–402 DE genes). Transcriptional differences between four pairs of brain regions showing highest dissimilarities of expression profiles are illustrated in Additional file 3: Figure S2. Complete list of DE genes for each pair of brain regions is shown in Additional file 1: Table S3. The majority of DE genes stratified by RNA type are protein coding, while 20 % come from non-coding RNAs (including lncRNA, lincRNA, antisense, processed transcripts, etc.) and 9.5 % from pseudogenes. This result reflects the more robust expression of numerous protein coding RNAs but highlights the potential importance of a small set of non-coding RNAs in the difference between brain regions.

**Table 1:** Differentially expression between tissues

BA22	BA24	BA46	Insula	Amygdala	Hippocampus	Post. putamen	Cerebellum	Raphae	
4	11	872	114	1281	1612	1801	4315	2876	BA10
	43	62	119	1761	1895	1891	3577	2791	BA22
		106	0	1123	1655	2252	4778	3548	BA24
			342	1114	1794	1193	2828	2176	BA46
				521	1322	1820	4415	3155	Insula
					251	797	3606	2256	Amygdala
						827	3206	1714	Hippocampus
							0	402	Post. putamen
								51	Cerebellum

The number of genes are listed found to be differentially expressed (FDR ≤ 0.05) between pairwise tissue comparisons. FDR correction was made for genes included in analysis based on detectability

### Differential RNA expression between smokers and non-smokers

We again performed differential gene expression analysis with edgeR which uses a Poisson model. With an analysis performed for each brain region, we searched for genes differentially expressed between smokers and nonsmokers. For differential expression analysis, we considered only RNAs with CPM (counts per million total reads) > 2 in every tissue sample used in a comparison. For any given comparison, this left 8000 to 14,000 genes for analysis. In all tissues except BA46 and raphae nuclei (one subject missing for each), 5 smokers and 5 nonsmokers were included. BA46 from one smoker displayed a highly variable expression profile but analysis with RUVseq was able to reduce the variability seen in this sample allowing for its inclusion. Under these experimental conditions, we did not expect to detect many transcriptome-wide significant differences, and therefore, also relied on previously identified candidate genes. As a result of the small sample size and tendency for batch effects, most regions had few to no genes with transcriptome-wide significance at either level, while 56 genes were identified as differentially expressed in BA46 with FDR ≤ 0.1, including several non-coding genes (RP11-294 K24.4, LINC00617, AC144521.1) and pseudogenes (RP11-768G7.1, GJA1P1). An additional 39 genes in BA22, 2 genes in the insula, and one gene in the raphae nucleus were significant with FDR ≤ 0.1 (see Additional file 1: Table S3 for all genes and FDR levels). Focusing on known nicotine related genes in BA46, 14 genes were significantly different with FDR ≤ 0.1, and 7 of these retained significance at FDR ≤ 0.05. In BA22, 6 out of 39 genes with FDR ≤ 0.1 were nicotine related. In raphae nuclei, a single significant gene was nicotine related (SEMA3C FDR = 0.05). All genes with FDR ≤ 0.1 are listed in Additional file 1: Table S4 and nicotine related genes are marked with an asterisk. Functional enrichment analysis with toppgene targeting genes relating BA46 to smoking found 13 genes related to “response to abiotic stimulus” (GO:0009628, FDR B&H = 4E-3), such as: *VEGFA, HIF3a*, *TP53BP2* and *IGFBP7*, 10 genes relating to “behavior” (GO:0007610, FDR B&H = 8e-3), such as: *CIART*, *GPR37* and *PTN*; 11 genes related to transmembrane transport (GO:0055085 FDR B&H = 3E-2) including: *ATP13A4*, *ATP1A2*, *SLC1A2* and *SLC1A2* (Table 2).

**Table 2:** Enriched GO terms for differentially expressed smoking related genes in BA46

Gene	Gene information	GO term
APOLD1	Apolipoprotein L domain containing 1	Response to abiotic stimulus
ATP13A4	ATPase type 13A4	Transmembrane transport
ATP1A2	ATPase, Na+/K+ transporting, alpha 2 polypeptide	Behavior / response to abiotic stimulus / transmembrane transport
CIART	Circadian associated repressor of transcription	Behavior
GJA1	Gap junction protein, alpha 1, 43 kDa	Behavior / response to abiotic stimulus / transmembrane transport
GPR37	G protein-coupled receptor 37 (endothelin receptor type B-like)	Behavior
HIF3A	Hypoxia inducible factor 3, alpha subunit	Response to abiotic stimulus
IGFBP7	Insulin-like growth factor binding protein 7	Response to abiotic stimulus
MLC1	Megalencephalic leukoencephalopathy with subcortical cysts 1	Response to abiotic stimulus / transmembrane transport
PLOD2	Procollagen-lysine, 2-oxoglutarate 5-dioxygenase 2	Response to abiotic stimulus
PREX2	Phosphatidylinositol-3,4,5-trisphosphate-dependent Rac exchange factor 2	Behavior
PTN	Pleiotrophin	Behavior
S1PR1	Sphingosine-1-phosphate receptor 1	Behavior
SDC2	Syndecan 2	Response to abiotic stimulus
SDC4	Syndecan 4	Response to abiotic stimulus
SLC14A1	Urea transporter,Kidd blood group	Transmembrane transport
SLC1A2	Glial high affinity glutamate transporter	Behavior / response to abiotic stimulus / transmembrane transport
SLC1A3	Glial high affinity glutamate transporter	Behavior / response to abiotic stimulus / transmembrane transport
SLC4A4	Sodium bicarbonate cotransporter	Transmembrane transport
SLC5A11	Sodium/inositol cotransporter	Transmembrane transport
SLC7A11	Anionic amino acid transporter light chain, xc- system	Transmembrane transport
SLCO1C1	Solute carrier organic anion transporter	Transmembrane transport
TP53BP2	Tumor protein p53 binding protein 2	Response to abiotic stimulus
VEGFA	Vascular endothelial growth factor A	Behavior / response to abiotic stimulus

Two GO terms were enriched from genes with significant (FDR ≤ 0.1) differential expression between smokers and nonsmokers in BA46

### Pathway analysis of RNAs with differential expression between smokers and non-smokers

To understand how the differentially expressed genes from BA46 relate to smoking and nicotine, we built a custom pathway with the Ingenuity Pathway Analysis (IPA) package. Differentially expressed genes were added to a custom pathway, together with the terms “nicotine,” “smoking,” and all smoking related molecules. Connections were made between differentially expressed genes and the additional molecules using default IPA options. A prominent pathway in this analysis focuses on *VEGFA*, with genes more than two links from *VEGFA* removed (Additional file 4: Figure S3). Differentially expressed RNAs involved in this pathway, highlighted in green, were all higher in non-smoker tissues (BA46).

### Analysis of RNA isoform expression across brain regions

**Fig. 4:**
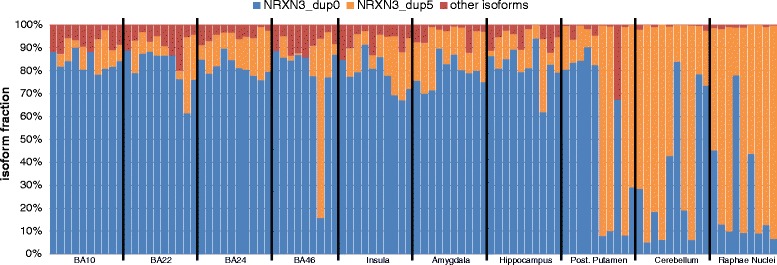
*NRXN3* isoform representation across brain region. *NRXN3* was found to be a gene with extreme differences in isoform representation. The top panel shows 5 isoforms annotated by RefSeq and the middle panel focuses on the difference between the major isoforms (Dup0 and Dup5). The bottom panel shows the representation of all isoforms as a fraction of the whole gene expression, combining Dup1 and Dup2 into “minor isoforms”. Dup0 is the major isoform in all Broadmann’s areas, insula, amygdala, and hippocampus. Dup5 is the major isoform in cerebellum and raphae nuclei. Samples from the posterior putamen are mixed

A majority of genes generate multiple RNA isoforms that often differ between tissues. To detect characteristic differences in brain region-selective expression, we ranked genes yielding isoform ratios with largest variability between regions (averages outside the expected 99 % confidence interval). Table 3 lists genes with the highest scores, while Fig. 4 highlights neurexin-3 (encoded by *NRXN3*) as an example. A complete record can be found in Additional file 1: Table S5 of the average isoform fraction of all genes considered across brain regions. The non-coding versions have at least one unique exon that does not overlap with coding versions of *NRXN3*. Alignment to these isoform-specific exons direct analysis of the expression levels. The raphae nuclei, cerebellum, and posterior putamen tend to express mostly the short *NRXN3* transcript dup5 isoform while other tissues express mostly full length NRXN3 dup0. The short isoform dup5 lacks a large portion of the 5′ end and likely supports different functions, annotated as non-coding RNA lacking known coding potential. The non-coding versions have at least one unique exon that does not overlap with coding versions of *NRXN3*. Alignment to these isoform-specific exons direct analysis of the expression levels.

**Table 3:** Genes with the most isoform variation between brain regions

Ranking	Gene	Isoform ranking score	Gencode ID	RNA type
1	GATS	9.0	ENSG00000160844.6	Protein
2	NRXN3	9.0	ENSG00000021645.13	Protein
3	R3HDM1	9.0	ENSG00000048991.12	Protein
4	RTN4	9.0	ENSG00000115310.13	Protein
5	BRWD1	8.7	ENSG00000185658.9	Protein
6	MLIP	8.7	ENSG00000146147.10	Protein
7	PART1	8.7	ENSG00000152931.7	Noncoding
8	PIK3R1	8.5	ENSG00000145675.10	Protein
9	POLR1D	8.5	ENSG00000186184.11	Protein
10	PVRL3	8.3	ENSG00000177707.6	Protein
11	SEPT8	8.3	ENSG00000164402.9	Protein
12	CEP85L	8.0	ENSG00000111860.9	Protein
13	DLG2	8.0	ENSG00000150672.12	Protein
14	ELMO1	8.0	ENSG00000155849.11	Protein
15	INPP5F	8.0	ENSG00000198825.7	Protein
16	MAGI1	8.0	ENSG00000151276.18	Protein
17	SYNPO	8.0	ENSG00000171992.8	Protein
18	WDR47	8.0	ENSG00000085433.11	Protein
19	KALRN	7.8	ENSG00000160145.10	Protein
20	GAS7	7.7	ENSG00000007237.13	Protein
21	GPM6B	7.7	ENSG00000046653.10	Protein
22	ANTXR1	7.5	ENSG00000169604.15	Protein
23	ATP5S	7.5	ENSG00000125375.10	Protein
24	DGKG	7.5	ENSG00000058866.10	Protein
25	DYNC1I2	7.5	ENSG00000077380.11	Protein
26	NTRK2	7.5	ENSG00000148053.11	Protein
27	PTER	7.5	ENSG00000165983.10	Protein
28	SLC29A2	7.5	ENSG00000174669.7	Protein
29	WNK1	7.5	ENSG00000060237.12	Protein

The top scoring genes are listed with variable isoform usage among brain regions. Order determined by comparing the average expression of each tissue to the average expression across all tissues and individuals. Genes with more tissue variation rank higher

Quantification of expression is sensitive to the read assignment method applied, such as the one used by Cufflinks, to distribute reads to expressed regions of isoforms over the whole gene. While Cufflinks probabilistically assign reads to isoforms and is widely accepted as an accurate expression quantification method, one could focus on local differences, i.e.*,* what exons are being included or excluded, and consider a custom set of isoforms based on expression within the working dataset. One option within cufflinks is to quantify the expression within the dataset and generate a gene annotation file based on that data; however, this approach can merge genes that should be kept separate. To test whether the results change when using only well expressed isoforms for quantification, we reran cufflinks with NRXN3_dup0 and NRXN3_dup5 as the only two isoforms for *NRXN3*. For those tissues with a high percentage of the full length *NRXN3*, 13 % of isoforms are expressed as dup5 from an alternative start site generating a shorter NRXN3 RNA. For the raphae nuclei and cerebellum, this percentage goes up to 65 %. When limiting the gene annotation set to dup5 and dup0 isoforms, most reads originally assigned to minor isoforms and dup4 were assigned to dup5, changing the isoform fractions marginally.

### SNP calling and allele-selective RNA expression, or allelic expression imbalance (AEI)

Several filters were employed to select SNPs contributing to a measure of allelic expression imbalance across a gene. Requiring a 95 % confidence level as the lower bound to the AEI ratio of SNPs within a gene for a given subject/region, an average of 24 RNAs displayed a robust signature of possible allelic expression imbalance (AEI) per region, with ~1600 genes represented across all regions and subjects (1.4 K protein coding, 177 non-coding, 22 pseudogenes). These results point to the presence of frequent regulatory variants affecting the expression of all RNA classes. Additional file 5: Figure S4 shows a scatter-plot of the average magnitude of the allelic RNA ratio compared to the average read depth for SNPs contained in the gene (with a twofold allelic RNA ratio above the 95 % CI as the cutoff). Genes with more extreme imbalanced ratios tend to have lower read depth, which decreases precision of measuring allele specific expression. On the basis of this graph, we chose an arbitrary read cutoff of 30 reads per SNP for the purpose of the present analysis.

With a stringent read filter of 30 reads per SNP and twofold AEI ratio, we detect 443 genes with likely AEI in any region such as Huntington’s Disease-associated: *ELMO1*, *NTRK2*, *WNK1* and associated with Schizophrenia: *NTRK2*, *PIK3R1* and *RTN4*. Table 4 contains the top 20 genes with AEI detected in any sample, sorted by magnitude of AEI ratios and by brain region. A complete listing is found in Additional file 1: Table S5. Most of these genes have detectable AEI in 1 tissue in 1 sample as detection of AEI depends on expression level. At the stringent 30 read filter, 6 genes were detected with AEI in more than one subject of the same brain regions, a finding likely associated with high minor allele frequency of a regulatory variants. Table 5 shows these 6 genes and results from eQTL and GWAS databases. Of these 6 genes, *LPAR1, PSD3,* and *GNAS* are associated with brain-related phenotypes (alcoholism, memory, and brain waves), and *LPAR1, PSD3, GNAS,* and *SRPK2* with eQTLs annotated in GTex or PheGenI in any human tissue (brain regions are not well represented in GTEx). It is apparent that these genes carry frequent regulatory variants with robust effects on RNA expression levels. We are currently developing mathematical and statistical methodologies for examining the landscape of allelic expression ratio to extract instances of less robust AEI.

**Table 4:** Top 20 AEI per tissue

	Amygdala	BA10	BA22	BA24	BA46	Cerebellum	Hippocampus	Insula	Post. putamen	Raphae nucleus
1	SNHG14	BCAP29	C7orf41	ATP6V1G2	SLC8A1	ERV3-1	RP11-785H5.1	CD24P4	CCT5	GSTA4
2	STON2	LMO7	ZNF91	ATP6V1G2-DDX39B	RCAN2	RP11-862 L9.3	RP11-785H5.2	TTTY14	lnc-SNURF-3	NDRG3
3	lnc-SNURF-3	NHP2L1	NHP2L1	SYNJ1	SPTBN1	CTD-2353 F22.1	DNAJA4	PILRB	SNHG14	RALGAPB
4	AFTPH	NUDT5	ZNF391	TXN2	LPHN3	NMNAT2	PDE1A	AC005592.2	PDXP	RBM26
5	UBE3A	RAB21	NGEF	PRPF8	FAM212B	GPRIN3	LANCL2	FGF1	SH3BP1	GPRIN3
6	PEG3	UBE3A	SENP2	FAM120A	PSD3	CHN1	DCP2	MAP1LC3B	SPP1	SOGA1
7	ZIM2	IL6ST	LRRC6	NHP2L1	AHSA1	SEPT3	C9orf72	POLR3F	ZEB1	NALCN
8	RP11-746 M1.1	UFM1	LPAR1	YWHAB	SERINC1	UBR3	USP47	RPL21P3	PYGB	FUT9
9	CELSR2	PDGFRA	RPS20	TAOK1	ANKS1B	GAS7	KIAA1549	CCDC103	YWHAG	RIMS2
10	AP1S1	ZEB1	ENO4	POMP	LINGO1	PLK2	NEFL	FAM187A	PBX3	NHP2L1
11	NHP2L1	NEO1	KIAA1598	RP11-269G24.3	ANK2	EPB41L1	PRRC2C	GFAP	MANBAL	NMNAT2
12	OPA1	TAF2	NECAB2	TANC2	AL391357.1	NEDD4L	STARD13	ARHGAP32	SV2B	SEPT3
13	WDFY3	AL391152.1	FAM107A	IGFBP5	DDOST	MAP2	CROCCP3	PCM1	SETD6	PPP3CA
14	MEF2A	CNGB3	CYFIP1	PPFIBP1	PINK1	YWHAG	EPHA7	PDE8B	KIF5C	USP24
15	LHFPL3	CPNE3	ARHGAP32	SPHKAP	PINK1-AS	lnc-GALNT2-1	AC010127.3	NRXN3	ECHS1	RP11-981G7.1
16	RPS6KA2	ATF6	SCP2	MYO5A	CTSB	AL691479.1	SCN1A	EIF2AK4	QKI	GAS7
17	CDC14B	NAV2-AS1	ANKS1B	GAS7	FMN2	CASC7	C1orf226	PRDM2	FAM13C	MAPK9
18	PRICKLE2	TNS3	PPP1R12B	PRKCB	DST	MBP	RP11-565P22.6	WDR41	ACIN1	RP11-463C8.4
19	RP11-129B22.1	ADNP2	SLC6A1	RP11-862 L9.3	--	NCL	AJAP1	ARCN1	FDFT1	ZNF91
20	ARHGAP32	EXOC5	APBA1	PCDH9	--	MAP1A	SRPK2	TPM1	MOCS1	APOL2

After restricting the list of genes with AEI to those with >30 reads per SNP averaged across gene, the list for each region is sorted by average AEI fold-change

**Table 5:** Genes with AEI in 2 samples in a region

Region	Gene	AEI ratio Avg ± S.D.	Avg SNPs per gene per sample	GTex eQTL	PheGenI eQTL	PheGenI association
BA10	AKAP12	3.7 ± 0.6	6			
BA22	LPAR1	3.3 ± 0.7	3	Whole blood	Brain cerebellum	Alcoholism
BA46	PSD3	3.4 ± 0.7	2		Brain pons	Memory
Cerebellum	GNAS	2.8 ± 0.4	2		Liver, lymphoblastoid	Brain waves
Hippocampus	SRPK2	3.6 ± 0.7	2	Whole blood, esophagus	Lymphoblastoid	
Raphaenucleus	PDE4DIP	3.4 ± 0.8	3			

Genes with allelic expression imbalance in 2 subjects in the same brain region after stringent filtering of 30 reads of coverage. Listed is the average and standard deviation of the AEI fold change for each gene and the average number of SNPs contributing to the AEI signal for each gene per sample. Genes were checked against public databases for eQTLs and SNP associations

### Gene expression confirmation

RNA-seq values of selected genes were orthogonally verified using Taq-Man qRT-PCR gene expression measurements. Real-time PCR was used to evaluate expression levels of three of the invariable genes applied to normalize the sequencing reads (AGO1, SPEN, SRSF11 each expressed at different FPKM levels) and also to re-evaluate the expression profiles of three nicotine related genes with variable expression (HIF3A, SLC1A3, NRXN3). Expression levels were measured by qRT PCR in all available brain regions of each sample. Using a log transformation, the overall pearson correlation of these measurements was 0.92. See Additional file 6: Figure S5 for a scatterplot of the comparison.

### Data sharing

The data supporting the results of this article are available in the GEO repository (accession ID: GSE68559 link: http://www.ncbi.nlm.nih.gov/geo/query/acc.cgi?token=kvaxwogwdxgjvwx&acc=GSE68559).

## Discussion

We present here the results for RNAseq analyses in ten human brain regions from ten subjects, five with a history of smoking and five controls. Owing to the preparation of sequencing libraries with both poly-*dT* and random hexamer primers, all RNA classes are detected, thereby, providing additional detail not available in previous studies of brain transcriptomes, such as transcript isoforms, non-coding RNAs, and allelic ratios as indicators of regulatory variants. A first analysis of differential gene expression between smokers and non-smokers was guided by previous results, as the number of subjects is limited for an independent analysis.

### Abundance and distribution of protein coding and non-coding RNAs

With both random hexamer and poly-*dT* priming, we covered all RNAs at least 200 bases long, regardless of poly-adenylation status. Wide-ranging transcription from a considerable portion of the genome has led to the discovery of tens of thousands of non-coding RNAs with diverse functions. Our results in human brain regions illustrate the robust expression of a large number of protein coding mRNAs compared to non-coding RNAs, even though only 1.2 % of the genome consists of coding exons. More protein coding genes are expressed across all 10 regions than non-coding RNAs and pseudogenes. This finding suggests lncRNA, pseudogenes, and processed transcripts are more region-specific than protein coding transcripts and could support distinct functions critical to specific tissues. Preliminary analysis not reported here shows that gene networks derived from the RNA expression patterns may be strengthened with the inclusion of non-coding RNAs with ncRNAs serving as relays in protein-coding RNA networks but further work is needed (unpublished results). The brain region RNAseq data provided here appear to be useful for network analyses and defining potential functions of ncRNAs.

### Transcriptome analysis of brain regions from smokers and controls

Our study was designed to complement previous transcriptome analyses, and to serve as a starting point for extended analysis of a larger cohort, or to study the isoform expression profiles of known candidate genes. The identification of differentially expressed genes, either from tissue to tissue comparisons or from smoker vs nonsmoker, highlights the effect of sequencing batch on gene expression, as a confounding factor. Regions from BA46 were sequenced in the same run and are best suited for comparisons within the region between smokers and nonsmokers. Conversely, the other nine regions were sequenced in one run for each subject separately, leading to a batch effects between regions. Without added normalization, these nine regions are best suited for tissue to tissue comparisons as they have the same batch biases. Here we have extracted those genes with similar expression across all regions, and in addition those that are invariant between subjects, employing a Shannon entropy-driven analysis. We propose that these genes can serve broadly as genes for normalization of RNAseq data acquired from heterogeneous tissues such as brain. Using these invariable genes with RUVseq to remove unwanted variation, we were able to overcome batch effects leading to enhanced difference between BA46 and other tissues, and yielding differential gene counts expected from biological similarity between brain regions.

A number of genes were differentially expressed between smokers and non-smokers, detectable mostly in brain region BA46, including WIF1, CX3CR1, and APOLD1. Using Ingenuity pathway analysis with nicotine as the central theme, several of the differentially expressed RNAs were found to connect directly or indirectly to VEGFA, which in turn connects to smoking pathways through VCAM1 and DRD2. VEGF is a growth factor involved in angiogenesis, vasculogenesis, and endothelial cell growth (UniProtKB/Swiss-Prot), while previous studies report various associations with nicotine and smoking. Cigarette smoke was found to reduce *VEGF* levels in human umbilical vein endothelial cells [48], whereas two other studies failed to detect a correlation between *VEGF* plasma levels and smoking status [49, 50]. Smoking was further associated with VEGF receptor expression [49] and abnormal endothelial function [50]. Moreover, VEGF may have a protective role in ischemia and stroke [51, 52], potentially counteracting the deleterious effects of smoking. Neuroprotective effects of VEGF under ischemia had been demonstrated in rat neurons [51]. Further studies are needed to follow up on the role of differentially expressed genes in smokers versus non-smokers.

### Differential expression of RNA isoforms across brain regions

Our RNAseq database is well suited to detect RNA isoforms that occur at nearly all gene loci. Here we have focused on an analysis of genes yielding isoforms with distinct distribution patterns between brain regions. We use neurexin-3, encoded by *NRXN3*, as one example of substantial differences between tissues. Whole gene expression of *NRXN3* in brain regions was robust, ranging from 20 to 43 FPKM. Raphae nuclei and cerebellum expressed more NRXN3_dup5 isoform while other tissues express higher amounts of NRXN3_dup0 full length mRNA. Samples from the posterior putamen show a mixture of either more dup5 or more dup0 isoforms, likely due to differences in cellular heterogeneity or genetic factors. Both isoforms are labeled as “noncoding” by RefSeq and “nonsense mediated decay” by ensembl suggesting it may play a role in reducing erroneous gene expression. As neither isoform is thought to express a protein, one must take isoform distribution across brain regions into account in biological studies to avoid erroneous conclusions. Looking at the exons unique to the dup0 and dup5 isoforms, not present in any coding version, it appears that both isoforms are robustly and differentially expressed between brain regions into RNA. Moreover, we identified AEI for NRXN3 RNA in three tissues —insula, amygdala, and BA46 (each for a different sample), while no eQTLs are listed for NRXN3 in GTEx; this may result from averaging all isoforms to yield a composite mRNA level. Our results can lead to the identification of regulatory variants in *NRXN3.*

Comparing average whole gene expression of NRXN3 for 4 brain regions (amygdala, BA24, cerebellum and hippocampus) present in both our data set and in the Genotype-Tissue Expression (GTEx) project, yielded a strong correlation with *r* = 0.92. In both datasets, NRXN3 is expressed in higher amounts in the cerebellum compared to other regions. For a direct comparison between the OSU and GTEx NRXN3 isoform expression, we adopted the transcript profile annotated in GTEx. In our dataset, the three most prominent isoforms were ENST00000428277.2, ENST00000555387.1, and ENST00000554738.1, while in the GTEx brain regions, ENST00000428277.2 accounts for approximately 80 % of NRXN3 mRNA compared to ~30 % in the OSU brains. This difference is likely due to library preparation and poly-A selection employed by GTEx. If we focus on the latter two isoforms, the ratio of these two isoforms favors ENST00000554738.1 in both GTEx and OSU in the cerebellum and favors ENST00000555387.1 in the other 3 overlapping regions. ENST00000554738.1 corresponds to NRXN3_dup5, and ENST00000555387.1 corresponds to NRXN3_dup0 confirming the results shown in Additional file 7: Figure S6.

### Allelic RNA expression

Using strict filtering criteria, we identified a group of genes with allelic expression imbalance in any sample from different brain regions. The stringent filters used here detect only the most striking signals of allelic expression imbalance. The combination of these parameters with large scale genotyping with GeneChip of the gDNA provides strong evidence a variant is truly heterozygous and that a finding of allelic RNA expression imbalance (AEI) is justified. This approach yielded a list of genes likely to be under regulatory influence where one copy of the gene is preferentially expressed. Given that only ten subjects were analyzed, the minor allele frequency of any regulatory variant can vary substantially over a broad range. However, this range is much narrower when 2 of 10 subjects display AEI for any given gene, implying a mean allele frequency of ~10 %. We detect 6 genes with AEI in more than one subject, indicating that a frequent regulatory variant is present with substantial effect on expression (Table 4). Four of these genes had been previously identified as containing eQTLs, i.e., variants that are associated with mRNA expression in various tissues, providing independent confirmation that these genes are under regulatory influence in other tissues. Three genes were found to be associated with clinical phenotypes in GWAS studies (Table 4). *LPAR1* encodes a receptor for lysophosphatidic acid and has been associated with alcoholism (rs509276, located upstream of *LPAR1*; *p* = 2.5e-5 in Collaborative Study on the Genetics of Alcoholism COGA [53]). *PSD3* (encoding Pleckstrin and SEC7 domains-containing protein 3) is a cancer risk gene that has been associated with memory functions (rs901732, intronic, *p* = 3.7e-8; rs1386687, intronic, *p* = 7.9e-6; Framingham Heart Study [54]). Lastly, *GNAS* (encoding the Gsα subunit of stimulatory G proteins) is a critical signaling molecule in the activation of adenylyl cyclase and has been associated with numerous disorders and phenotypes (see OMIM). A detailed analysis of all cases of AEI is ongoing.

## Conclusions

RNA sequencing identifies distinct and consistent differences in gene expression between brain regions. Non-coding RNAs are also differentially expressed between brain regions and may play a role in regulation of gene expression and functional differentiation of the different brain areas. Smoking affects coding and non-coding transcript expression in BA46 in a number of genes related to nicotine exposure. The purpose of this report was to provide an overview of the data source created by sequencing 10 brain regions of 10 subjects. We present here patterns of expression of various types of RNA, differential expression between tissues, differences in the expression of RNA isoforms, and detection of allelic expression analysis identifying genes under regulatory genetic influence.

## Additional files

Additional file 1:
**Table S1.** Sequencing and mapping statistics. Listed are the sequencing details and mapping statistics for the presented data. Sequencing sets varied in the number of lanes per run, sequencing technology, and inclusion of paired reads. For sets with replicate runs, the number of lanes for each run and the sequencer technology are separated by slashes. Some replicate runs were generated under different sequencing conditions (SOLiD5500 technology vs wildfire technology) and this is indicated when appropriate. **Table S2.** Normalizing genes identified by mathematical modeling. 2a Top 200 invariable genes. Lists top 20 genes found to be constant across subjects and tissues. 2b Top 200 stable genes. Lists top 20 genes found to be constant across subjects, but different between tissues. **Table S3.** Differentially expressed genes between brain regions. Lists all significantly (FDR ≤ 0.05) differentially expressed genes between any two brain regions identified by RUVseq after removing unwanted variation using invariable genes. **Table S4.** Genes differentially expressed between smokers and nonsmokers. Lists the genes found to be differentially expressed between smokers and nonsmokers (FDR ≤ 0.1). Genes previously implicated in nicotine or smoking are marked with an asterisk. **Table S5.** Isoform fraction across brain regions. Lists the average isoform fraction across brain regions for genes passing expression level filters. Genes were considered when having ≥ 5 FPKM in at least 40 libraries. To detect genes generating different isoform patterns in different brain regions we sorted genes with multiple isoform by the number of tissues with an isoform average outside the 99 % confidence interval generated from all samples providing a simple measure of how much individual regions deviated from the average. **Table S6.** Genes with AEI. Lists the genes identified with potential allelic expression imbalance. Genes were required to have 2+ SNPs and an average coverage of 30 reads for those SNPs. (DOCX 99 kb) Additional file 2: Figure S1 Average expression of invariable genes in 4 GTEx brains. Shows the variation of 200 invariable genes expressed in 4 GTEx brain regions. (PDF 189 kb) Additional file 3: Figure S2 Visualization of the results of differential expression analysis for four pairs of brain regions with highest differences in gene expression. log fold-change (x-axis) and corresponding –log FDR (y-axis) are shown for each gene. Genes differentially expressed (FDR < 0.05) are marked in red and top five DE genes for each pair are indicated. (TIF 915 kb) Additional file 4: Figure S3 Pathway relating VEGF and other differentially expressed genes to smoking. Displays a pathway generated by Ingenuity Pathway Analysis using genes differentially expressed between smokers and nonsmokers in BA46 that connect to smoking and smoking related molecules. Differentially expressed genes are highlighted in green and smoking related molecules are outlined in purple. (PDF 80 kb) Additional file 5: Figure S4 Plot of AEI ratio versus read depth across SNP. Displays the average magnitude of the allelic expression fold-change of a gene for a particular sample/region compared to the average read depth for SNPs in the gene. SNPs with low coverage tend to have more extreme AEI. (PDF 211 kb) Additional file 6: Figure S5 Confirmation of expression levels by qRTPCR. qRTPCR was performed to correlate the FPKM of 3 invariably expressed (AGO1, SPEN, SRSF11) and 3 nicotine candidate genes (HIF3A, SLC1A3, NRXN3) for nearly all 100 samples. qRTPCR was quantified using the ∆Δct method and using the average of the three invariable genes for normalization. Across brain regions, we can correlate the RTPCR measured expression with the RNAseq measured expression. After a log transformation, the overall Pearson correlation is 0.92. (PDF 173 kb) Additional file 7: Figure S6 Comparison of NRXN3 isoform representation between OSU and GTEx brains. The ratio of the blue and orange isoforms favors ENST00000554738.1 in both GTEx and OSU in the cerebellum and favors ENST00000555387.1 in the other 3 overlapping regions. ENST00000554738.1 corresponds to NRXN3_dup5, and ENST00000555387.1 corresponds to NRXN3_dup0. The protein coding version ENST00000428277.2 is highly represented in GTEx brains likely due to poly-A selection. (PDF 176 kb)

## Abbreviations

AEI: Allelic expression imbalance
BA: Broadmann area
CNS: Central nervous system
CPM: Counts per million
eQTL: Expression quantitative trait loci
FDR: False discovery rate
FPKM: Fragments Per Kilobase per Million reads
GO: Gene ontology
GTEx: Genotype-tissue expression project
GWAS: Genome-wide association study
IPA: Ingenuity pathway analysis
IUPAC: International Union of Pure and Applied Chemistry ambiguity codes for SNPs
lincRNA: Long intergenic noncoding RNA
lncRNA: Long noncoding RNA
ncRNA: Non-coding RNA
OMIM: Online Mendelian inheritance in man
PCR: Polymerase chain reaction
RPKM: Reads Per Kilobase per Million reads
SNP: Single nucleotide polymorphism
