# Supplementary material for: RNA sequencing of transcriptomes in human brain regions: protein-coding and non-coding RNAs, isoforms and alleles
Source: BMC Genomics. 2015 Nov 23;16:990. doi: 10.1186/s12864-015-2207-8 (PMC4657279; doi:10.1186/s12864-015-2207-8)
Supplement: Additional file 1: — Table S1. Sequencing and mapping statistics. Listed are the sequencing details and mapping statistics for the presented data. Sequencing sets varied in the number of lanes per run, sequencing technology, and inclusion of paired reads. For sets with replicate runs, the number of lanes for each run and the sequencer technology are separated by slashes. Some replicate runs were generated under different sequencing conditions (SOLiD5500 technology vs wildfire technology) and this is indicated when appropriate. Table S2. Normalizing genes identified by mathematical modeling. 2a Top 200 invariable genes. Lists top 20 genes found to be constant across subjects and tissues. 2b Top 200 stable genes. Lists top 20 genes found to be constant across subjects, but different between tissues. Table S3. Differentially expressed genes between brain regions. Lists all significantly (FDR ≤ 0.05) differentially expressed genes between any two brain regions identified by RUVseq after removing unwanted variation using invariable genes. Table S4. Genes differentially expressed between smokers and nonsmokers. Lists the genes found to be differentially expressed between smokers and nonsmokers (FDR ≤ 0.1). Genes previously implicated in nicotine or smoking are marked with an asterisk. Table S5. Isoform fraction across brain regions. Lists the average isoform fraction across brain regions for genes passing expression level filters. Genes were considered when having ≥ 5 FPKM in at least 40 libraries. To detect genes generating different isoform patterns in different brain regions we sorted genes with multiple isoform by the number of tissues with an isoform average outside the 99 % confidence interval generated from all samples providing a simple measure of how much individual regions deviated from the average. Table S6. Genes with AEI. Lists the genes identified with potential allelic expression imbalance. Genes were required to have 2+ SNPs and an average coverage of 30 reads for those SNPs. [file 12864_2015_2207_MOESM1_ESM.docx]

Supplementary table 1

| **Set** | **Lanes** | **Sequencer** | **Generated** | **Avg. Fragments** | **Forward Mapping** | **Reverse Mapping** |
| --- | --- | --- | --- | --- | --- | --- |
| BA46 | 3 | wildfire | paired | 58,498,666 | 71% | 46% |
| MB100 | 3/1 | wildfire | paired/single | 32,198,926 | 75% | 19% |
| MB11 | 6/6/1 | SOLiD5500/wildfire | paired/single | 27,872,652 | 84% | 67% |
| MB147 | 1/2/2 | wildfire | paired | 45,605,077 | 75% | 65% |
| MB148 | 4 | wildfire | paired | 116,546,911 | 75% | 67% |
| MB151 | 6 | SOLiD5500 | paired | 32,624,912 | 64% | 79% |
| MB160 | 1/1 | wildfire | paired | 26,573,801 | 69% | 48% |
| MB197 | 2 | wildfire | paired | 49,457,583 | 74% | 52% |
| MB202 | 3 | wildfire | paired | 45,894,505 | 71% | 41% |
| MB52 | 6/6 | SOLiD5500 | paired/single | 42,932,922 | 79% | 66% |
| MB59 | 3 | wildfire | single | 70,047,907 | 72% | -- |

Supplementary table 2a

| Gencode id | Gene name | RNA type |
| --- | --- | --- |
| ENSG00000151498.7 | ACAD8 | protein |
| ENSG00000101126.11 | ADNP | protein |
| ENSG00000092847.6 | AGO1 | protein |
| ENSG00000164022.12 | AIMP1 | protein |
| ENSG00000131503.16 | ANKHD1 | protein |
| ENSG00000176915.10 | ANKLE2 | protein |
| ENSG00000132466.13 | ANKRD17 | protein |
| ENSG00000021776.6 | AQR | protein |
| ENSG00000107863.12 | ARHGAP21 | protein |
| ENSG00000049618.16 | ARID1B | protein |
| ENSG00000196440.7 | ARMCX4 | protein |
| ENSG00000116539.6 | ASH1L | protein |
| ENSG00000171681.8 | ATF7IP | protein |
| ENSG00000166454.5 | ATMIN | protein |
| ENSG00000146776.10 | ATXN7L1 | protein |
| ENSG00000122507.16 | BBS9 | protein |
| ENSG00000182831.7 | C16orf72 | protein |
| ENSG00000157181.10 | C1orf27 | protein |
| ENSG00000163608.10 | C3orf17 | protein |
| ENSG00000205765.4 | C5orf51 | protein |
| ENSG00000012822.11 | CALCOCO1 | protein |
| ENSG00000110395.4 | CBL | protein |
| ENSG00000181982.13 | CCDC149 | protein |
| ENSG00000186166.4 | CCDC84 | protein |
| ENSG00000129315.5 | CCNT1 | protein |
| ENSG00000065883.10 | CDK13 | protein |
| ENSG00000101639.13 | CEP192 | protein |
| ENSG00000198707.10 | CEP290 | protein |
| ENSG00000115561.10 | CHMP3 | protein |
| ENSG00000130779.15 | CLIP1 | protein |
| ENSG00000176444.14 | CLK2 | protein |
| ENSG00000158435.3 | CNOT11 | protein |
| ENSG00000080802.14 | CNOT4 | protein |
| ENSG00000183513.4 | COA5 | protein |
| ENSG00000113742.8 | CPEB4 | protein |
| ENSG00000110925.2 | CSRNP2 | protein |
| ENSG00000268205.1 | CTC-444N24.11 | noncoding |
| ENSG00000163257.6 | DCAF16 | protein |
| ENSG00000139990.13 | DCAF5 | protein |
| ENSG00000132153.10 | DHX30 | protein |
| ENSG00000108406.5 | DHX40 | protein |
| ENSG00000101191.12 | DIDO1 | protein |
| ENSG00000160305.13 | DIP2A | protein |
| ENSG00000151240.11 | DIP2C | protein |
| ENSG00000083097.10 | DOPEY1 | protein |
| ENSG00000157540.14 | DYRK1A | protein |
| ENSG00000148730.6 | EIF4EBP2 | protein |
| ENSG00000127463.9 | EMC1 | protein |
| ENSG00000100393.9 | EP300 | protein |
| ENSG00000152223.8 | EPG5 | protein |
| ENSG00000139163.11 | ETNK1 | protein |
| ENSG00000081177.14 | EXD2 | protein |
| ENSG00000171824.9 | EXOSC10 | protein |
| ENSG00000158161.11 | EYA3 | protein |
| ENSG00000113194.8 | FAF2 | protein |
| ENSG00000182518.9 | FAM104B | protein |
| ENSG00000112584.8 | FAM120B | protein |
| ENSG00000082269.12 | FAM135A | protein |
| ENSG00000145780.6 | FEM1C | protein |
| ENSG00000162613.12 | FUBP1 | protein |
| ENSG00000172461.6 | FUT9 | protein |
| ENSG00000143614.7 | GATAD2B | protein |
| ENSG00000257218.1 | GATC | protein |
| ENSG00000101193.6 | GID8 | protein |
| ENSG00000016864.12 | GLT8D1 | protein |
| ENSG00000112624.8 | GLTSCR1L | protein |
| ENSG00000144674.12 | GOLGA4 | protein |
| ENSG00000116580.13 | GON4L | protein |
| ENSG00000108587.10 | GOSR1 | protein |
| ENSG00000186566.7 | GPATCH8 | protein |
| ENSG00000068400.9 | GRIPAP1 | protein |
| ENSG00000110768.7 | GTF2H1 | protein |
| ENSG00000232561.2 | GTF2IP1 | pseudogene |
| ENSG00000172432.14 | GTPBP2 | protein |
| ENSG00000231074.4 | HCG18 | noncoding |
| ENSG00000128731.11 | HERC2 | protein |
| ENSG00000122557.5 | HERPUD2 | protein |
| ENSG00000186834.2 | HEXIM1 | protein |
| ENSG00000165102.10 | HGSNAT | protein |
| ENSG00000110422.7 | HIPK3 | protein |
| ENSG00000159267.9 | HLCS | protein |
| ENSG00000100281.9 | HMGXB4 | protein |
| ENSG00000104824.11 | HNRNPL | protein |
| ENSG00000102786.10 | INTS6 | protein |
| ENSG00000068745.10 | IP6K2 | protein |
| ENSG00000083168.5 | KAT6A | protein |
| ENSG00000100578.10 | KIAA0586 | protein |
| ENSG00000110318.9 | KIAA1377 | protein |
| ENSG00000164323.8 | KIAA1430 | protein |
| ENSG00000176542.5 | KIAA2018 | protein |
| ENSG00000183354.7 | KIAA2026 | protein |
| ENSG00000131023.8 | LATS1 | protein |
| ENSG00000258441.1 | LINC00641 | noncoding |
| ENSG00000185621.7 | LMLN | protein |
| ENSG00000136802.7 | LRRC8A | protein |
| ENSG00000204406.7 | MBD5 | protein |
| ENSG00000053524.7 | MCF2L2 | protein |
| ENSG00000169057.14 | MECP2 | protein |
| ENSG00000125686.7 | MED1 | protein |
| ENSG00000108788.7 | MLX | protein |
| ENSG00000119950.16 | MXI1 | protein |
| ENSG00000102921.3 | N4BP1 | protein |
| ENSG00000140396.8 | NCOA2 | protein |
| ENSG00000198646.9 | NCOA6 | protein |
| ENSG00000114904.8 | NEK4 | protein |
| ENSG00000072736.14 | NFATC3 | protein |
| ENSG00000086102.14 | NFX1 | protein |
| ENSG00000001167.10 | NFYA | protein |
| ENSG00000164190.12 | NIPBL | protein |
| ENSG00000162408.10 | NOL9 | protein |
| ENSG00000101361.10 | NOP56 | protein |
| ENSG00000149308.11 | NPAT | protein |
| ENSG00000243716.6 | NPIPB5 | protein |
| ENSG00000196993.4 | NPIPB9 | protein |
| ENSG00000133961.15 | NUMB | protein |
| ENSG00000124789.7 | NUP153 | protein |
| ENSG00000110713.11 | NUP98 | protein |
| ENSG00000166889.13 | PATL1 | protein |
| ENSG00000185619.13 | PCGF3 | protein |
| ENSG00000203880.6 | PCMTD2 | protein |
| ENSG00000077684.11 | PHF17 | protein |
| ENSG00000118482.7 | PHF3 | protein |
| ENSG00000146247.13 | PHIP | protein |
| ENSG00000040199.14 | PHLPP2 | protein |
| ENSG00000187953.6 | PMS2CL | pseudogene |
| ENSG00000058600.11 | POLR3E | protein |
| ENSG00000110851.7 | PRDM4 | protein |
| ENSG00000117523.11 | PRRC2C | protein |
| ENSG00000121390.13 | PSPC1 | protein |
| ENSG00000224597.4 | PTCHD3P1 | noncoding |
| ENSG00000185129.4 | PURA | protein |
| ENSG00000129472.8 | RAB2B | protein |
| ENSG00000137502.5 | RAB30 | protein |
| ENSG00000136828.14 | RALGPS1 | protein |
| ENSG00000122257.14 | RBBP6 | protein |
| ENSG00000181827.10 | RFX7 | protein |
| ENSG00000080345.12 | RIF1 | protein |
| ENSG00000164197.7 | RNF180 | protein |
| ENSG00000137075.13 | RNF38 | protein |
| ENSG00000185946.11 | RNPC3 | protein |
| ENSG00000246596.2 | RP11-1277A3.2 | pseudogene |
| ENSG00000188971.3 | RP11-427H3.2 | noncoding |
| ENSG00000225784.5 | RP11-592B15.4 | pseudogene |
| ENSG00000255455.2 | RP11-890B15.3 | noncoding |
| ENSG00000163125.11 | RPRD2 | protein |
| ENSG00000204130.8 | RUFY2 | protein |
| ENSG00000136715.13 | SAP130 | protein |
| ENSG00000156304.10 | SCAF4 | protein |
| ENSG00000113615.8 | SEC24A | protein |
| ENSG00000138802.7 | SEC24B | protein |
| ENSG00000181555.15 | SETD2 | protein |
| ENSG00000107957.12 | SH3PXD2A | protein |
| ENSG00000205060.6 | SLC35B4 | protein |
| ENSG00000116698.16 | SMG7 | protein |
| ENSG00000065526.6 | SPEN | protein |
| ENSG00000080603.12 | SRCAP | protein |
| ENSG00000116754.9 | SRSF11 | protein |
| ENSG00000119760.11 | SUPT7L | protein |
| ENSG00000157625.11 | TAB3 | protein |
| ENSG00000125875.9 | TBC1D20 | protein |
| ENSG00000197226.8 | TBC1D9B | protein |
| ENSG00000168769.8 | TET2 | protein |
| ENSG00000152291.9 | TGOLN2 | protein |
| ENSG00000158604.10 | TMED4 | protein |
| ENSG00000143337.13 | TOR1AIP1 | protein |
| ENSG00000169905.7 | TOR1AIP2 | protein |
| ENSG00000182606.10 | TRAK1 | protein |
| ENSG00000100815.8 | TRIP11 | protein |
| ENSG00000130338.8 | TULP4 | protein |
| ENSG00000165006.9 | UBAP1 | protein |
| ENSG00000107341.4 | UBE2R2 | protein |
| ENSG00000110344.5 | UBE4A | protein |
| ENSG00000157741.9 | UBN2 | protein |
| ENSG00000024048.6 | UBR2 | protein |
| ENSG00000169062.10 | UPF3A | protein |
| ENSG00000109189.7 | USP46 | protein |
| ENSG00000183878.11 | UTY | protein |
| ENSG00000166887.11 | VPS39 | protein |
| ENSG00000095787.16 | WAC | protein |
| ENSG00000140153.13 | WDR20 | protein |
| ENSG00000109685.13 | WHSC1 | protein |
| ENSG00000066422.4 | ZBTB11 | protein |
| ENSG00000174282.7 | ZBTB4 | protein |
| ENSG00000177888.7 | ZBTB41 | protein |
| ENSG00000188177.9 | ZC3H6 | protein |
| ENSG00000133858.11 | ZFC3H1 | protein |
| ENSG00000165156.10 | ZHX1 | protein |
| ENSG00000174306.17 | ZHX3 | protein |
| ENSG00000198315.6 | ZKSCAN8 | protein |
| ENSG00000152926.9 | ZNF117 | protein |
| ENSG00000010244.12 | ZNF207 | protein |
| ENSG00000213096.5 | ZNF254 | protein |
| ENSG00000188994.8 | ZNF292 | protein |
| ENSG00000197714.4 | ZNF460 | protein |
| ENSG00000186020.8 | ZNF529 | protein |
| ENSG00000196967.6 | ZNF585A | protein |
| ENSG00000245680.5 | ZNF585B | protein |
| ENSG00000121864.5 | ZNF639 | protein |
| ENSG00000197372.5 | ZNF675 | protein |
| ENSG00000133624.9 | ZNF767 | pseudogene |

Supplementary table 2b

| Gencode id | Gene name | RNA type |
| --- | --- | --- |
| ENSG00000115977.14 | AAK1 | protein |
| ENSG00000136754.12 | ABI1 | protein |
| ENSG00000135503.8 | ACVR1B | protein |
| ENSG00000072364.8 | AFF4 | protein |
| ENSG00000092847.6 | AGO1 | protein |
| ENSG00000162433.10 | AK4 | protein |
| ENSG00000101901.6 | ALG13 | protein |
| ENSG00000181754.6 | AMIGO1 | protein |
| ENSG00000167522.10 | ANKRD11 | protein |
| ENSG00000132466.13 | ANKRD17 | protein |
| ENSG00000124198.8 | ARFGEF2 | protein |
| ENSG00000145819.11 | ARHGAP26 | protein |
| ENSG00000160007.13 | ARHGAP35 | protein |
| ENSG00000196914.4 | ARHGEF12 | protein |
| ENSG00000102606.13 | ARHGEF7 | protein |
| ENSG00000116539.6 | ASH1L | protein |
| ENSG00000152092.10 | ASTN1 | protein |
| ENSG00000115966.12 | ATF2 | protein |
| ENSG00000171681.8 | ATF7IP | protein |
| ENSG00000088812.13 | ATRN | protein |
| ENSG00000204842.9 | ATXN2 | protein |
| ENSG00000158470.5 | B4GALT5 | protein |
| ENSG00000009954.6 | BAZ1B | protein |
| ENSG00000129473.5 | BCL2L2 | protein |
| ENSG00000145734.14 | BDP1 | protein |
| ENSG00000204217.8 | BMPR2 | protein |
| ENSG00000204256.8 | BRD2 | protein |
| ENSG00000096070.15 | BRPF3 | protein |
| ENSG00000166167.13 | BTRC | protein |
| ENSG00000182831.7 | C16orf72 | protein |
| ENSG00000088854.11 | C20orf194 | protein |
| ENSG00000135932.6 | CAB39 | protein |
| ENSG00000108349.10 | CASC3 | protein |
| ENSG00000110395.4 | CBL | protein |
| ENSG00000181982.13 | CCDC149 | protein |
| ENSG00000176749.4 | CDK5R1 | protein |
| ENSG00000173575.13 | CHD2 | protein |
| ENSG00000100888.8 | CHD8 | protein |
| ENSG00000080802.14 | CNOT4 | protein |
| ENSG00000113742.8 | CPEB4 | protein |
| ENSG00000149532.11 | CPSF7 | protein |
| ENSG00000099942.8 | CRKL | protein |
| ENSG00000259806.2 | CTD-2196E14.4 | noncoding |
| ENSG00000260482.1 | CTD-2196E14.9 | noncoding |
| ENSG00000198561.8 | CTNND1 | protein |
| ENSG00000055130.11 | CUL1 | protein |
| ENSG00000139990.13 | DCAF5 | protein |
| ENSG00000145833.11 | DDX46 | protein |
| ENSG00000110367.7 | DDX6 | protein |
| ENSG00000101191.12 | DIDO1 | protein |
| ENSG00000151240.11 | DIP2C | protein |
| ENSG00000088538.12 | DOCK3 | protein |
| ENSG00000157540.14 | DYRK1A | protein |
| ENSG00000115504.10 | EHBP1 | protein |
| ENSG00000148730.6 | EIF4EBP2 | protein |
| ENSG00000075151.15 | EIF4G3 | protein |
| ENSG00000100393.9 | EP300 | protein |
| ENSG00000204442.2 | FAM155A | protein |
| ENSG00000116199.7 | FAM20B | protein |
| ENSG00000100350.10 | FOXRED2 | protein |
| ENSG00000109536.7 | FRG1 | protein |
| ENSG00000143614.7 | GATAD2B | protein |
| ENSG00000145990.6 | GFOD1 | protein |
| ENSG00000101193.6 | GID8 | protein |
| ENSG00000204120.10 | GIGYF2 | protein |
| ENSG00000111670.10 | GNPTAB | protein |
| ENSG00000116580.13 | GON4L | protein |
| ENSG00000082701.10 | GSK3B | protein |
| ENSG00000077809.8 | GTF2I | protein |
| ENSG00000232561.2 | GTF2IP1 | pseudogene |
| ENSG00000128731.11 | HERC2 | protein |
| ENSG00000186834.2 | HEXIM1 | protein |
| ENSG00000163349.17 | HIPK1 | protein |
| ENSG00000110422.7 | HIPK3 | protein |
| ENSG00000095951.12 | HIVEP1 | protein |
| ENSG00000181666.13 | HKR1 | protein |
| ENSG00000165119.14 | HNRNPK | protein |
| ENSG00000086758.11 | HUWE1 | protein |
| ENSG00000143195.8 | ILDR2 | protein |
| ENSG00000104331.4 | IMPAD1 | protein |
| ENSG00000152409.8 | JMY | protein |
| ENSG00000156650.8 | KAT6B | protein |
| ENSG00000176595.3 | KBTBD11 | protein |
| ENSG00000176542.5 | KIAA2018 | protein |
| ENSG00000134313.10 | KIDINS220 | protein |
| ENSG00000101350.6 | KIF3B | protein |
| ENSG00000178502.5 | KLHL11 | protein |
| ENSG00000167548.10 | KMT2D | protein |
| ENSG00000107929.9 | LARP4B | protein |
| ENSG00000116977.14 | LGALS8 | protein |
| ENSG00000258441.1 | LINC00641 | noncoding |
| ENSG00000070018.4 | LRP6 | protein |
| ENSG00000136802.7 | LRRC8A | protein |
| ENSG00000127603.19 | MACF1 | protein |
| ENSG00000168175.9 | MAPK1IP1L | protein |
| ENSG00000169057.14 | MECP2 | protein |
| ENSG00000108510.5 | MED13 | protein |
| ENSG00000063322.9 | MED29 | protein |
| ENSG00000068305.13 | MEF2A | protein |
| ENSG00000130396.16 | MLLT4 | protein |
| ENSG00000188895.6 | MSL1 | protein |
| ENSG00000104643.5 | MTMR9 | protein |
| ENSG00000116984.8 | MTR | protein |
| ENSG00000197535.10 | MYO5A | protein |
| ENSG00000161048.7 | NAPEPLD | protein |
| ENSG00000140396.8 | NCOA2 | protein |
| ENSG00000198646.9 | NCOA6 | protein |
| ENSG00000102908.16 | NFAT5 | protein |
| ENSG00000169992.5 | NLGN2 | protein |
| ENSG00000091129.15 | NRCAM | protein |
| ENSG00000165671.14 | NSD1 | protein |
| ENSG00000074590.9 | NUAK1 | protein |
| ENSG00000069275.12 | NUCKS1 | protein |
| ENSG00000198585.7 | NUDT16 | protein |
| ENSG00000272325.1 | NUDT3 | protein |
| ENSG00000100836.6 | PABPN1 | protein |
| ENSG00000090060.13 | PAPOLA | protein |
| ENSG00000166889.13 | PATL1 | protein |
| ENSG00000203880.6 | PCMTD2 | protein |
| ENSG00000113448.12 | PDE4D | protein |
| ENSG00000087495.12 | PHACTR3 | protein |
| ENSG00000118482.7 | PHF3 | protein |
| ENSG00000141720.7 | PIP4K2B | protein |
| ENSG00000132424.10 | PNISR | protein |
| ENSG00000116731.18 | PRDM2 | protein |
| ENSG00000142875.15 | PRKACB | protein |
| ENSG00000183530.9 | PRR14L | protein |
| ENSG00000117523.11 | PRRC2C | protein |
| ENSG00000132300.14 | PTCD3 | protein |
| ENSG00000179912.15 | R3HDM2 | protein |
| ENSG00000124209.3 | RAB22A | protein |
| ENSG00000129472.8 | RAB2B | protein |
| ENSG00000010017.9 | RANBP9 | protein |
| ENSG00000173166.13 | RAPH1 | protein |
| ENSG00000122257.14 | RBBP6 | protein |
| ENSG00000119707.9 | RBM25 | protein |
| ENSG00000184863.6 | RBM33 | protein |
| ENSG00000003756.12 | RBM5 | protein |
| ENSG00000167257.6 | RNF214 | protein |
| ENSG00000218418.2 | RP11-296E7.1 | pseudogene |
| ENSG00000163125.11 | RPRD2 | protein |
| ENSG00000156304.10 | SCAF4 | protein |
| ENSG00000138802.7 | SEC24B | protein |
| ENSG00000214765.4 | SEPT7P2 | pseudogene |
| ENSG00000145391.8 | SETD7 | protein |
| ENSG00000162105.11 | SHANK2 | protein |
| ENSG00000198053.7 | SIRPA | protein |
| ENSG00000101307.11 | SIRPB1 | protein |
| ENSG00000160785.9 | SLC25A44 | protein |
| ENSG00000205060.6 | SLC35B4 | protein |
| ENSG00000080503.15 | SMARCA2 | protein |
| ENSG00000116698.16 | SMG7 | protein |
| ENSG00000065526.6 | SPEN | protein |
| ENSG00000106723.12 | SPIN1 | protein |
| ENSG00000115306.11 | SPTBN1 | protein |
| ENSG00000167881.10 | SRP68 | protein |
| ENSG00000133226.12 | SRRM1 | protein |
| ENSG00000167978.12 | SRRM2 | protein |
| ENSG00000116754.9 | SRSF11 | protein |
| ENSG00000141298.13 | SSH2 | protein |
| ENSG00000173320.5 | STOX2 | protein |
| ENSG00000117758.9 | STX12 | protein |
| ENSG00000196235.9 | SUPT5H | protein |
| ENSG00000157625.11 | TAB3 | protein |
| ENSG00000135090.9 | TAOK3 | protein |
| ENSG00000125875.9 | TBC1D20 | protein |
| ENSG00000131374.10 | TBC1D5 | protein |
| ENSG00000100207.14 | TCF20 | protein |
| ENSG00000054118.9 | THRAP3 | protein |
| ENSG00000198586.9 | TLK1 | protein |
| ENSG00000205269.4 | TMEM170B | protein |
| ENSG00000128872.5 | TMOD2 | protein |
| ENSG00000067369.9 | TP53BP1 | protein |
| ENSG00000143549.15 | TPM3 | protein |
| ENSG00000182606.10 | TRAK1 | protein |
| ENSG00000071575.7 | TRIB2 | protein |
| ENSG00000107341.4 | UBE2R2 | protein |
| ENSG00000111647.8 | UHRF1BP1L | protein |
| ENSG00000170832.8 | USP32 | protein |
| ENSG00000115464.10 | USP34 | protein |
| ENSG00000109189.7 | USP46 | protein |
| ENSG00000138592.9 | USP8 | protein |
| ENSG00000162923.10 | WDR26 | protein |
| ENSG00000164091.7 | WDR82 | protein |
| ENSG00000109685.13 | WHSC1 | protein |
| ENSG00000171475.9 | WIPF2 | protein |
| ENSG00000130227.12 | XPO7 | protein |
| ENSG00000119596.13 | YLPM1 | protein |
| ENSG00000163374.15 | YY1AP1 | protein |
| ENSG00000174282.7 | ZBTB4 | protein |
| ENSG00000058673.11 | ZC3H11A | protein |
| ENSG00000123200.12 | ZC3H13 | protein |
| ENSG00000204186.3 | ZDBF2 | protein |
| ENSG00000148516.17 | ZEB1 | protein |
| ENSG00000131381.8 | ZFYVE20 | protein |
| ENSG00000165156.10 | ZHX1 | protein |
| ENSG00000215421.5 | ZNF407 | protein |
| ENSG00000196967.6 | ZNF585A | protein |
| ENSG00000245680.5 | ZNF585B | protein |
| ENSG00000183309.7 | ZNF623 | protein |

Supplementary table 4

| **Brain region** | **Short gene name** | **RNA type** | **Gene ID** | **log_2_FC** | **FDR** |
| --- | --- | --- | --- | --- | --- |
| BA22 | AKAP5 | protein | ENSG00000179841.7 | 0.9 | 0.06 |
| BA22 | B2M | protein | ENSG00000166710.13 | -1 | 0.1 |
| BA22 | CAPZA1 | protein | ENSG00000116489.8 | -0.9 | 0.09 |
| BA22 | CCND1* | protein | ENSG00000110092.3 | -2 | 0.1 |
| BA22 | CD44* | protein | ENSG00000026508.12 | -2 | 0.08 |
| BA22 | CDK6* | protein | ENSG00000105810.5 | -1.2 | 0.04 |
| BA22 | CPS1 | protein | ENSG00000021826.10 | -1.5 | 0.03 |
| BA22 | CTGF* | protein | ENSG00000118523.5 | -2.3 | 0.02 |
| BA22 | DCBLD2 | protein | ENSG00000057019.11 | -1.6 | 0.01 |
| BA22 | DCDC2 | protein | ENSG00000146038.7 | -2.1 | 0.03 |
| BA22 | EIF3E | protein | ENSG00000104408.5 | -0.9 | 0.07 |
| BA22 | FGF5 | protein | ENSG00000138675.12 | -1.9 | 0.09 |
| BA22 | FSTL1 | protein | ENSG00000163430.5 | -1.8 | 0.03 |
| BA22 | HOMER1 | protein | ENSG00000152413.10 | 0.9 | 0.09 |
| BA22 | ITGB1 | protein | ENSG00000150093.14 | -2 | 0.09 |
| BA22 | KCTD9 | protein | ENSG00000104756.11 | -1 | 0.09 |
| BA22 | KIF15* | protein | ENSG00000163808.12 | -2.2 | 0.01 |
| BA22 | MGST1 | protein | ENSG00000008394.8 | -1.8 | 0.04 |
| BA22 | MT-ATP8 | protein | ENSG00000228253.1 | -1.6 | 0.09 |
| BA22 | MTATP8P2 | pseudogene | ENSG00000229604.2 | -1.5 | 0.1 |
| BA22 | MT-ND4 | protein | ENSG00000198886.2 | -1 | 0.09 |
| BA22 | MTND5P13 | pseudogene | ENSG00000250169.1 | -1.3 | 0.01 |
| BA22 | NPM1 | protein | ENSG00000181163.9 | -1 | 0.04 |
| BA22 | NPM1P27 | pseudogene | ENSG00000249353.2 | -1.1 | 0.09 |
| BA22 | PDCD10 | protein | ENSG00000114209.10 | -1.4 | 0.09 |
| BA22 | PLG | protein | ENSG00000122194.13 | -1.1 | 0.09 |
| BA22 | RAI14 | protein | ENSG00000039560.9 | -1.3 | 0.09 |
| BA22 | RP11-262D11.2 | pseudogene | ENSG00000225471.2 | -1.3 | 0.03 |
| BA22 | RP11-442A13.1 | pseudogene | ENSG00000234782.2 | -1.4 | 0.05 |
| BA22 | RP1-83M4.2 | pseudogene | ENSG00000217027.1 | -1.4 | 0.04 |
| BA22 | RPL34 | protein | ENSG00000109475.12 | -1.1 | 0.09 |
| BA22 | SEC11A | protein | ENSG00000140612.9 | -1.5 | 0.06 |
| BA22 | SLC22A3* | protein | ENSG00000146477.4 | -1.3 | 0.06 |
| BA22 | SMS | protein | ENSG00000102172.11 | -0.9 | 0.09 |
| BA22 | STK38 | protein | ENSG00000112079.8 | -0.9 | 0.09 |
| BA22 | TPM4 | protein | ENSG00000167460.10 | -1.2 | 0.1 |
| BA22 | TPT1 | protein | ENSG00000133112.12 | -1.4 | 0.03 |
| BA22 | UACA | protein | ENSG00000137831.10 | -1.9 | 0.02 |
| BA22 | ZFAS1 | noncoding | ENSG00000177410.8 | -1.5 | 0.01 |
| BA46 | AC144521.1 | noncoding | ENSG00000228956.3 | -0.8 | 0.05 |
| BA46 | ACSS3 | protein | ENSG00000111058.3 | -0.7 | 0.07 |
| BA46 | APOLD1 | protein | ENSG00000178878.7 | -1.5 | 0.001 |
| BA46 | ATP13A4 | protein | ENSG00000127249.10 | -1 | 0.04 |
| BA46 | ATP1A2 | protein | ENSG00000018625.10 | -0.8 | 0.06 |
| BA46 | BBOX1 | protein | ENSG00000129151.4 | -0.9 | 0.05 |
| BA46 | C1orf51 | protein | ENSG00000159208.11 | -0.9 | 0.07 |
| BA46 | CDC42EP4 | protein | ENSG00000179604.8 | -0.8 | 0.01 |
| BA46 | CLDND1 | protein | ENSG00000080822.12 | 0.9 | 0.08 |
| BA46 | CX3CR1 | protein | ENSG00000168329.9 | 1.2 | 0.000004 |
| BA46 | CXCL14* | protein | ENSG00000145824.8 | -0.9 | 0.01 |
| BA46 | CYP4F11* | protein | ENSG00000171903.11 | -0.9 | 0.03 |
| BA46 | EFEMP1* | protein | ENSG00000115380.14 | -1 | 0.1 |
| BA46 | ETNPPL | protein | ENSG00000164089.4 | -1.3 | 0.02 |
| BA46 | GADD45B* | protein | ENSG00000099860.4 | -1.4 | 0.07 |
| BA46 | GJA1 | protein | ENSG00000152661.7 | -1 | 0.01 |
| BA46 | GJA1P1 | pseudogene | ENSG00000176857.4 | -0.9 | 0.07 |
| BA46 | GJB6* | protein | ENSG00000121742.11 | -1.4 | 0.01 |
| BA46 | GLUL* | protein | ENSG00000135821.11 | -0.9 | 0.07 |
| BA46 | GPR125 | protein | ENSG00000152990.9 | -1 | 0.02 |
| BA46 | GPR37* | protein | ENSG00000170775.2 | 0.9 | 0.08 |
| BA46 | HIF3A | protein | ENSG00000124440.11 | -1 | 0.06 |
| BA46 | IGFBP7 | protein | ENSG00000163453.7 | -1.4 | 0.001 |
| BA46 | LINC00617 | noncoding | ENSG00000250366.2 | 0.8 | 0.07 |
| BA46 | MAP3K6 | protein | ENSG00000142733.10 | -0.9 | 0.05 |
| BA46 | MLC1 | protein | ENSG00000100427.11 | -0.8 | 0.03 |
| BA46 | MRPL33 | protein | ENSG00000243147.3 | 1 | 0.03 |
| BA46 | MT2A* | protein | ENSG00000125148.6 | -1.3 | 0.01 |
| BA46 | PAMR1 | protein | ENSG00000149090.7 | -0.9 | 0.07 |
| BA46 | PARD3B | protein | ENSG00000116117.13 | -0.8 | 0.1 |
| BA46 | PLOD2 | protein | ENSG00000152952.7 | -0.8 | 0.05 |
| BA46 | PLTP | protein | ENSG00000100979.10 | -1 | 0.05 |
| BA46 | PON2 | protein | ENSG00000105854.8 | -0.9 | 0.06 |
| BA46 | PPAP2B* | protein | ENSG00000162407.8 | -1 | 0.07 |
| BA46 | PREX2 | protein | ENSG00000046889.14 | -0.8 | 0.04 |
| BA46 | PTN | protein | ENSG00000105894.7 | -0.8 | 0.07 |
| BA46 | RANBP3L | protein | ENSG00000164188.4 | -1.2 | 0.004 |
| BA46 | RIT2 | protein | ENSG00000152214.8 | 0.7 | 0.1 |
| BA46 | RP11-294K24.4 | noncoding | ENSG00000260800.1 | -0.9 | 0.02 |
| BA46 | RP11-768G7.1 | pseudogene | ENSG00000240393.1 | -0.8 | 0.06 |
| BA46 | S1PR1 | protein | ENSG00000170989.8 | -1 | 0.01 |
| BA46 | SDC2 | protein | ENSG00000169439.7 | -0.8 | 0.1 |
| BA46 | SDC4 | protein | ENSG00000124145.5 | -1 | 0.04 |
| BA46 | SLC14A1* | protein | ENSG00000141469.12 | -1.7 | 0.02 |
| BA46 | SLC1A2* | protein | ENSG00000110436.7 | -1.3 | 0.01 |
| BA46 | SLC1A3 | protein | ENSG00000079215.9 | -1.6 | 0.004 |
| BA46 | SLC4A4* | protein | ENSG00000080493.9 | -1 | 0.02 |
| BA46 | SLC5A11 | protein | ENSG00000158865.8 | 1 | 0.03 |
| BA46 | SLC7A11* | protein | ENSG00000151012.9 | -0.9 | 0.07 |
| BA46 | SLCO1C1 | protein | ENSG00000139155.4 | -1.1 | 0.004 |
| BA46 | STON2 | protein | ENSG00000140022.5 | -0.8 | 0.07 |
| BA46 | TIMP3 | protein | ENSG00000100234.11 | -0.8 | 0.09 |
| BA46 | TP53BP2 | protein | ENSG00000143514.12 | -0.8 | 0.1 |
| BA46 | VEGFA* | protein | ENSG00000112715.16 | -0.9 | 0.07 |
| BA46 | WIF1 | protein | ENSG00000156076.5 | -1.8 | 0.000001 |
| BA46 | ZFP36 | protein | ENSG00000128016.4 | -2.1 | 0.07 |
| insula | MGST1 | protein | ENSG00000008394.8 | -1.7 | 0.06 |
| insula | PLG | protein | ENSG00000122194.13 | -2.5 | 0.06 |
| raphae | SEMA3C* | protein | ENSG00000075223.9 | -2 | 0.05 |

Supplementary table 5

| **region** | **gencode_gene_name** | **Avg±S.D.** | **Avg SNPs per gene per sample** | **Number of Samples Meeting Filter** |
| --- | --- | --- | --- | --- |
| BA10 | AKAP12 | 3.7±0.6 | 6 | 2 |
| cerebellum | GNAS | 2.8±0.4 | 2 | 2 |
| BA22 | LPAR1 | 3.3±0.7 | 3 | 2 |
| raphaenucleus | PDE4DIP | 3.3±0.8 | 3 | 2 |
| BA46 | PSD3 | 3.4±0.7 | 2 | 2 |
| hippocampus | SRPK2 | 3.9±0.9 | 2 | 2 |
| hippocampus | ABHD2 | 3.7±1.2 | 3 | 1 |
| amygdala | ABI2 | 2.3±0.2 | 2 | 1 |
| BA10 | AC004076.9 | 3.4±1.1 | 3 | 1 |
| insula | AC005592.2 | 4.3±0.5 | 2 | 1 |
| hippocampus | AC010127.3 | 4.2±1.8 | 4 | 1 |
| postputamen | AC024560.3 | 2.2±0.1 | 2 | 1 |
| postputamen | ACIN1 | 3.4±0.8 | 2 | 1 |
| raphaenucleus | ADARB1 | 3.1±0.7 | 3 | 1 |
| hippocampus | ADCYAP1R1 | 3.7±0.3 | 2 | 1 |
| BA10 | ADI1 | 4.4±0.8 | 3 | 1 |
| BA10 | ADNP2 | 5.0±1.0 | 2 | 1 |
| amygdala | AFTPH | 5.3±0.4 | 2 | 1 |
| BA46 | AHSA1 | 3.3±0.8 | 3 | 1 |
| hippocampus | AJAP1 | 4.0±1.7 | 5 | 1 |
| raphaenucleus | AKAP12 | 2.9±1.4 | 10 | 1 |
| BA24 | AKAP6 | 2.2±0.0 | 2 | 1 |
| BA10 | AL391152.1 | 5.4±1.1 | 4 | 1 |
| BA46 | AL391357.1 | 2.7±0.3 | 3 | 1 |
| cerebellum | AL691479.1 | 3.3±0.7 | 2 | 1 |
| BA46 | ANK2 | 2.8±0.5 | 6 | 1 |
| amygdala | ANK3 | 2.4±0.1 | 2 | 1 |
| BA24 | ANKRD12 | 2.2±0.1 | 2 | 1 |
| BA22 | ANKS1B | 2.6±0.3 | 5 | 1 |
| BA46 | ANKS1B | 3.3±0.8 | 2 | 1 |
| hippocampus | ANKS1B | 3.8±1.3 | 2 | 1 |
| amygdala | AP1S1 | 3.7±0.1 | 2 | 1 |
| BA22 | APBA1 | 2.4±0.3 | 2 | 1 |
| raphaenucleus | APOL2 | 3.7±1.1 | 3 | 1 |
| insula | ARCN1 | 3.0±0.7 | 3 | 1 |
| BA24 | ARHGAP24 | 3.0±0.3 | 2 | 1 |
| amygdala | ARHGAP32 | 3.1±0.6 | 2 | 1 |
| BA22 | ARHGAP32 | 2.8±0.3 | 2 | 1 |
| cerebellum | ARHGAP32 | 2.7±0.1 | 2 | 1 |
| insula | ARHGAP32 | 3.6±1.0 | 2 | 1 |
| BA10 | ARL6IP5 | 3.1±0.9 | 4 | 1 |
| hippocampus | ASIC2 | 2.6±1.0 | 16 | 1 |
| BA10 | ATF6 | 5.0±2.2 | 5 | 1 |
| BA10 | ATG10 | 2.6±0.1 | 2 | 1 |
| hippocampus | ATP1A2 | 2.2±0.0 | 3 | 1 |
| hippocampus | ATP5S | 3.4±0.4 | 2 | 1 |
| BA10 | ATP6V1E1 | 3.8±0.3 | 2 | 1 |
| BA24 | ATP6V1G2 | 7.0±3.6 | 2 | 1 |
| BA24 | ATP6V1G2-DDX39B | 7.0±3.6 | 2 | 1 |
| hippocampus | ATP9B | 3.0±0.3 | 2 | 1 |
| BA24 | ATRN | 3.1±0.3 | 2 | 1 |
| BA24 | ATRNL1 | 2.3±0.1 | 2 | 1 |
| BA24 | BACE1 | 2.7±0.1 | 2 | 1 |
| raphaenucleus | BAI3 | 3.4±2.9 | 18 | 1 |
| BA10 | BCAP29 | 7.5±4.0 | 4 | 1 |
| BA10 | BDP1 | 4.4±1.4 | 8 | 1 |
| cerebellum | BIRC6 | 2.2±0.1 | 2 | 1 |
| BA10 | BTRC | 2.6±0.1 | 2 | 1 |
| hippocampus | C16orf72 | 2.8±0.4 | 2 | 1 |
| hippocampus | C1orf226 | 4.2±1.6 | 3 | 1 |
| raphaenucleus | C1orf61 | 2.8±0.6 | 3 | 1 |
| BA10 | C2CD2 | 3.4±1.3 | 5 | 1 |
| hippocampus | C5orf22 | 2.5±0.3 | 3 | 1 |
| BA22 | C7orf41 | 9.0±2.9 | 3 | 1 |
| BA24 | C7orf41 | 2.5±0.5 | 4 | 1 |
| hippocampus | C9orf72 | 4.7±1.4 | 2 | 1 |
| amygdala | CACNG8 | 2.2±0.2 | 3 | 1 |
| raphaenucleus | CALN1 | 3.6±1.9 | 7 | 1 |
| hippocampus | CAMK4 | 2.7±0.4 | 2 | 1 |
| insula | CAP1 | 2.6±0.1 | 2 | 1 |
| cerebellum | CASC7 | 3.3±0.3 | 2 | 1 |
| raphaenucleus | CBLB | 3.1±0.5 | 3 | 1 |
| insula | CCDC103 | 3.6±0.1 | 2 | 1 |
| BA10 | CCDC91 | 2.6±0.6 | 5 | 1 |
| postputamen | CCT5 | 7.3±3.2 | 2 | 1 |
| insula | CD24P4 | 6.4±2.8 | 2 | 1 |
| hippocampus | CD46 | 3.9±1.3 | 2 | 1 |
| postputamen | CD46 | 2.8±0.5 | 4 | 1 |
| amygdala | CDC14B | 3.3±0.9 | 2 | 1 |
| cerebellum | CDH2 | 2.5±0.3 | 3 | 1 |
| hippocampus | CELF2 | 2.6±0.4 | 2 | 1 |
| amygdala | CELSR2 | 3.8±0.3 | 2 | 1 |
| insula | CELSR2 | 2.4±0.1 | 2 | 1 |
| amygdala | CHGB | 2.6±0.4 | 2 | 1 |
| hippocampus | CHGB | 3.2±0.3 | 2 | 1 |
| cerebellum | CHN1 | 5.0±1.9 | 5 | 1 |
| BA10 | CNGB3 | 5.3±2.8 | 3 | 1 |
| cerebellum | CNTN2 | 2.8±0.4 | 2 | 1 |
| hippocampus | CNTN2 | 2.3±0.2 | 2 | 1 |
| BA10 | COA5 | 3.0±0.4 | 2 | 1 |
| hippocampus | COG5 | 3.3±0.1 | 2 | 1 |
| BA10 | CPNE3 | 5.3±2.8 | 3 | 1 |
| hippocampus | CROCCP3 | 4.4±1.2 | 2 | 1 |
| BA24 | CSMD1 | 3.8±1.1 | 2 | 1 |
| BA10 | CSRNP3 | 4.1±0.1 | 2 | 1 |
| BA24 | CTB-55O6.12 | 2.3±0.0 | 2 | 1 |
| cerebellum | CTC-203F4.1 | 2.3±0.1 | 2 | 1 |
| postputamen | CTC-454I21.3 | 3.0±0.5 | 3 | 1 |
| hippocampus | CTC-513N18.7 | 2.3±0.1 | 2 | 1 |
| postputamen | CTD-2176I21.2 | 2.1±0.0 | 2 | 1 |
| cerebellum | CTD-2353F22.1 | 5.8±2.3 | 2 | 1 |
| BA46 | CTSB | 2.5±0.2 | 3 | 1 |
| BA10 | CWF19L2 | 4.0±1.4 | 2 | 1 |
| BA22 | CYFIP1 | 2.8±0.6 | 3 | 1 |
| cerebellum | DCAKD | 2.5±0.0 | 2 | 1 |
| hippocampus | DCP2 | 4.7±1.8 | 2 | 1 |
| postputamen | DDAH1 | 2.3±0.1 | 3 | 1 |
| BA10 | DDN | 2.3±0.0 | 2 | 1 |
| BA46 | DDOST | 2.7±0.3 | 3 | 1 |
| raphaenucleus | DEAF1 | 3.4±0.1 | 2 | 1 |
| BA10 | DLG1 | 3.0±0.4 | 2 | 1 |
| BA24 | DLG2 | 2.3±0.1 | 2 | 1 |
| hippocampus | DLG2 | 2.6±1.4 | 27 | 1 |
| cerebellum | DNAJA1 | 3.0±0.5 | 2 | 1 |
| hippocampus | DNAJA4 | 5.6±2.3 | 2 | 1 |
| raphaenucleus | DPP6 | 3.1±1.9 | 22 | 1 |
| BA24 | DPYSL2 | 3.3±1.3 | 4 | 1 |
| hippocampus | DROSHA | 2.4±0.3 | 3 | 1 |
| BA46 | DST | 2.2±0.1 | 2 | 1 |
| BA10 | DUSP8 | 2.8±0.4 | 2 | 1 |
| postputamen | ECHS1 | 3.5±0.7 | 2 | 1 |
| insula | EIF2AK4 | 3.3±0.2 | 2 | 1 |
| BA10 | EMC4 | 3.5±0.2 | 2 | 1 |
| BA24 | ENAH | 2.6±0.4 | 2 | 1 |
| BA22 | ENO4 | 3.1±0.6 | 2 | 1 |
| hippocampus | ENO4 | 3.8±0.6 | 2 | 1 |
| cerebellum | EPB41L1 | 4.2±1.0 | 3 | 1 |
| hippocampus | EPHA7 | 4.3±1.0 | 2 | 1 |
| cerebellum | ERV3-1 | 9.7±2.8 | 2 | 1 |
| BA10 | EXOC5 | 4.9±1.5 | 5 | 1 |
| hippocampus | FABP3 | 2.5±0.1 | 2 | 1 |
| BA22 | FAM107A | 2.8±0.6 | 2 | 1 |
| cerebellum | FAM107A | 2.7±0.4 | 3 | 1 |
| BA24 | FAM120A | 5.1±1.3 | 2 | 1 |
| postputamen | FAM120B | 2.6±0.3 | 2 | 1 |
| postputamen | FAM13C | 3.5±0.8 | 2 | 1 |
| BA10 | FAM171B | 2.3±0.2 | 2 | 1 |
| insula | FAM171B | 2.5±0.1 | 2 | 1 |
| insula | FAM187A | 3.6±0.1 | 2 | 1 |
| BA46 | FAM212B | 3.4±0.8 | 2 | 1 |
| hippocampus | FBXO7 | 3.9±0.6 | 2 | 1 |
| postputamen | FDFT1 | 3.4±1.3 | 4 | 1 |
| insula | FGF1 | 4.3±0.5 | 2 | 1 |
| BA46 | FMN2 | 2.4±0.0 | 2 | 1 |
| hippocampus | FMNL2 | 3.5±2.0 | 8 | 1 |
| hippocampus | FUT9 | 3.0±1.3 | 7 | 1 |
| raphaenucleus | FUT9 | 4.9±3.1 | 11 | 1 |
| hippocampus | GABRA1 | 2.6±0.1 | 2 | 1 |
| BA24 | GAS7 | 3.9±1.7 | 7 | 1 |
| cerebellum | GAS7 | 4.6±2.5 | 6 | 1 |
| raphaenucleus | GAS7 | 4.2±1.3 | 5 | 1 |
| raphaenucleus | GATM | 2.4±0.0 | 2 | 1 |
| insula | GFAP | 3.6±0.1 | 2 | 1 |
| BA10 | GIT2 | 3.8±2.0 | 6 | 1 |
| raphaenucleus | GIT2 | 3.4±1.4 | 5 | 1 |
| BA10 | GNG2 | 2.9±0.7 | 3 | 1 |
| hippocampus | GOLGB1 | 3.2±0.4 | 2 | 1 |
| raphaenucleus | GOLGB1 | 3.0±0.7 | 2 | 1 |
| BA24 | GOT1 | 3.4±0.9 | 3 | 1 |
| raphaenucleus | GOT1 | 3.1±0.1 | 3 | 1 |
| amygdala | GOT2 | 2.2±0.1 | 3 | 1 |
| BA10 | GOT2 | 2.6±0.3 | 3 | 1 |
| BA24 | GPD1L | 2.9±0.5 | 2 | 1 |
| cerebellum | GPRIN3 | 5.6±2.6 | 6 | 1 |
| raphaenucleus | GPRIN3 | 5.3±1.3 | 5 | 1 |
| raphaenucleus | GSTA4 | 6.5±2.6 | 2 | 1 |
| BA10 | GTF3C4 | 3.9±1.2 | 3 | 1 |
| cerebellum | H2AFY | 2.3±0.1 | 3 | 1 |
| BA24 | HABP4 | 2.6±0.4 | 2 | 1 |
| raphaenucleus | HABP4 | 3.7±0.3 | 2 | 1 |
| postputamen | HEATR5B | 3.1±0.4 | 2 | 1 |
| insula | HEPACAM | 2.5±0.1 | 2 | 1 |
| BA10 | HIVEP1 | 2.8±0.3 | 2 | 1 |
| BA10 | HOOK3 | 3.3±0.6 | 2 | 1 |
| BA24 | HSPH1 | 2.2±0.0 | 2 | 1 |
| hippocampus | HTT | 2.5±0.2 | 2 | 1 |
| BA24 | IGFBP5 | 4.2±1.2 | 3 | 1 |
| BA10 | IL6ST | 5.6±0.8 | 2 | 1 |
| BA10 | IPO5 | 3.5±0.6 | 2 | 1 |
| BA22 | ISCU | 2.1±0.0 | 2 | 1 |
| hippocampus | ITM2C | 2.7±0.3 | 3 | 1 |
| postputamen | JAK1 | 2.7±0.1 | 2 | 1 |
| hippocampus | KCNAB2 | 2.4±0.2 | 2 | 1 |
| hippocampus | KCNMA1 | 3.6±2.5 | 12 | 1 |
| hippocampus | KCNQ3 | 2.6±0.9 | 16 | 1 |
| BA10 | KCNQ5 | 3.0±1.9 | 13 | 1 |
| BA10 | KCTD1 | 3.6±1.1 | 2 | 1 |
| hippocampus | KDM4C | 2.3±0.0 | 2 | 1 |
| hippocampus | KIAA1549 | 4.7±2.9 | 5 | 1 |
| BA22 | KIAA1551 | 2.4±0.2 | 2 | 1 |
| BA22 | KIAA1598 | 3.1±0.6 | 2 | 1 |
| hippocampus | KIAA1598 | 3.8±0.6 | 2 | 1 |
| raphaenucleus | KIAA1737 | 3.4±1.2 | 6 | 1 |
| raphaenucleus | KIAA2026 | 3.5±0.7 | 2 | 1 |
| hippocampus | KIF1A | 2.7±0.6 | 3 | 1 |
| BA24 | KIF1B | 3.4±0.1 | 2 | 1 |
| hippocampus | KIF1B | 2.8±0.5 | 2 | 1 |
| cerebellum | KIF3B | 2.1±0.0 | 2 | 1 |
| BA24 | KIF3C | 3.4±0.2 | 2 | 1 |
| postputamen | KIF3C | 2.2±0.1 | 3 | 1 |
| hippocampus | KIF5A | 2.6±0.4 | 2 | 1 |
| amygdala | KIF5C | 2.9±0.4 | 3 | 1 |
| postputamen | KIF5C | 3.5±1.4 | 4 | 1 |
| postputamen | KLHL42 | 2.8±1.0 | 6 | 1 |
| raphaenucleus | KMT2D | 2.2±0.0 | 2 | 1 |
| hippocampus | KMT2E | 3.3±0.4 | 2 | 1 |
| hippocampus | LANCL2 | 4.7±1.3 | 3 | 1 |
| BA10 | LARS | 4.8±1.6 | 5 | 1 |
| amygdala | LHFPL3 | 3.4±0.8 | 2 | 1 |
| BA46 | LINGO1 | 3.1±0.7 | 3 | 1 |
| BA10 | LMO7 | 7.3±1.4 | 2 | 1 |
| cerebellum | lnc-GALNT2-1 | 3.3±0.7 | 2 | 1 |
| amygdala | lnc-SNURF-3 | 5.3±1.8 | 3 | 1 |
| postputamen | lnc-SNURF-3 | 5.6±2.6 | 3 | 1 |
| hippocampus | LNPEP | 2.7±0.2 | 2 | 1 |
| BA24 | LPHN1 | 2.3±0.0 | 2 | 1 |
| BA46 | LPHN3 | 3.5±0.7 | 2 | 1 |
| BA22 | LRRC6 | 3.5±0.7 | 2 | 1 |
| BA10 | LRRN1 | 2.8±0.9 | 6 | 1 |
| hippocampus | LRRTM2 | 2.6±0.4 | 2 | 1 |
| raphaenucleus | LYST | 3.7±1.5 | 5 | 1 |
| BA10 | MAGI2-IT1 | 4.3±0.6 | 2 | 1 |
| postputamen | MANBAL | 4.3±0.2 | 2 | 1 |
| BA24 | MANSC1 | 2.7±0.3 | 3 | 1 |
| cerebellum | MAP1A | 3.2±0.6 | 4 | 1 |
| raphaenucleus | MAP1B | 2.1±0.0 | 2 | 1 |
| insula | MAP1LC3B | 4.0±0.9 | 2 | 1 |
| cerebellum | MAP2 | 3.6±2.0 | 7 | 1 |
| BA22 | MAPK4 | 2.2±0.0 | 2 | 1 |
| cerebellum | MAPK9 | 2.4±0.3 | 3 | 1 |
| raphaenucleus | MAPK9 | 3.9±1.5 | 5 | 1 |
| BA24 | MAST3 | 2.3±0.1 | 2 | 1 |
| cerebellum | MBP | 3.3±1.4 | 7 | 1 |
| cerebellum | MDC1 | 2.6±0.1 | 2 | 1 |
| cerebellum | MDC1-AS1 | 2.6±0.1 | 2 | 1 |
| BA10 | MED31 | 4.7±1.9 | 2 | 1 |
| amygdala | MEF2A | 3.4±1.2 | 6 | 1 |
| hippocampus | MIR7-3HG | 3.7±1.6 | 4 | 1 |
| hippocampus | MKL2 | 3.1±0.4 | 2 | 1 |
| postputamen | MOCS1 | 3.2±0.3 | 2 | 1 |
| hippocampus | MRAS | 2.4±0.1 | 2 | 1 |
| cerebellum | MRFAP1 | 2.1±0.0 | 2 | 1 |
| BA10 | MRPS14 | 3.9±1.8 | 4 | 1 |
| raphaenucleus | MTUS1 | 3.3±1.8 | 11 | 1 |
| BA24 | MYO5A | 4.0±0.6 | 3 | 1 |
| insula | N4BP2L2 | 2.4±0.2 | 3 | 1 |
| raphaenucleus | NALCN | 5.0±2.4 | 3 | 1 |
| BA10 | NAV2-AS1 | 5.0±1.9 | 2 | 1 |
| insula | NCDN | 2.3±0.2 | 2 | 1 |
| cerebellum | NCL | 3.2±0.7 | 2 | 1 |
| hippocampus | NCL | 2.7±0.1 | 2 | 1 |
| amygdala | NDRG3 | 2.4±0.0 | 2 | 1 |
| raphaenucleus | NDRG3 | 6.2±0.6 | 2 | 1 |
| BA22 | NECAB2 | 3.0±0.6 | 2 | 1 |
| cerebellum | NEDD4L | 3.6±0.6 | 2 | 1 |
| BA10 | NEFL | 3.0±0.8 | 3 | 1 |
| hippocampus | NEFL | 4.6±1.2 | 2 | 1 |
| BA10 | NEO1 | 5.4±0.8 | 2 | 1 |
| insula | NFIA | 2.7±0.1 | 2 | 1 |
| BA22 | NGEF | 4.3±1.1 | 2 | 1 |
| amygdala | NHP2L1 | 3.6±1.1 | 4 | 1 |
| BA10 | NHP2L1 | 6.5±2.7 | 4 | 1 |
| BA22 | NHP2L1 | 6.1±2.9 | 4 | 1 |
| BA24 | NHP2L1 | 5.0±2.5 | 3 | 1 |
| raphaenucleus | NHP2L1 | 4.7±1.3 | 4 | 1 |
| cerebellum | NMNAT2 | 5.7±2.5 | 2 | 1 |
| raphaenucleus | NMNAT2 | 4.7±1.6 | 2 | 1 |
| BA10 | NOLC1 | 4.5±1.1 | 2 | 1 |
| postputamen | NPY | 2.5±0.1 | 2 | 1 |
| BA10 | NRIP3 | 2.7±0.4 | 2 | 1 |
| raphaenucleus | NRIP3 | 2.6±0.4 | 2 | 1 |
| BA24 | NRXN1 | 2.6±0.7 | 9 | 1 |
| hippocampus | NRXN1 | 2.5±1.5 | 42 | 1 |
| raphaenucleus | NRXN1 | 3.7±2.6 | 17 | 1 |
| insula | NRXN3 | 3.5±0.1 | 2 | 1 |
| hippocampus | NUCKS1 | 2.3±0.1 | 2 | 1 |
| raphaenucleus | NUCKS1 | 2.9±0.3 | 3 | 1 |
| BA10 | NUDT5 | 6.3±0.1 | 2 | 1 |
| raphaenucleus | OGFRL1 | 3.7±0.9 | 3 | 1 |
| postputamen | OLFM1 | 3.1±0.5 | 2 | 1 |
| amygdala | OPA1 | 3.5±1.0 | 3 | 1 |
| BA10 | ORMDL1 | 3.3±0.6 | 2 | 1 |
| amygdala | PACRGL | 3.0±0.5 | 3 | 1 |
| postputamen | PBX3 | 4.4±1.1 | 2 | 1 |
| BA24 | PCDH9 | 3.8±0.3 | 2 | 1 |
| raphaenucleus | PCGF3 | 2.7±0.5 | 4 | 1 |
| postputamen | PCLO | 2.6±0.1 | 3 | 1 |
| insula | PCM1 | 3.6±0.6 | 4 | 1 |
| hippocampus | PDE1A | 5.2±3.5 | 6 | 1 |
| amygdala | PDE4DIP | 3.0±0.7 | 3 | 1 |
| hippocampus | PDE4DIP | 3.7±0.0 | 2 | 1 |
| insula | PDE8B | 3.5±0.9 | 4 | 1 |
| BA10 | PDGFRA | 5.5±0.0 | 2 | 1 |
| BA10 | PDHX | 3.7±1.5 | 4 | 1 |
| postputamen | PDXP | 5.0±0.4 | 2 | 1 |
| amygdala | PEG3 | 4.1±1.5 | 3 | 1 |
| cerebellum | PHF17 | 2.5±0.3 | 2 | 1 |
| postputamen | PHF17 | 3.2±0.8 | 2 | 1 |
| insula | PI4KB | 2.8±0.2 | 2 | 1 |
| BA24 | PIK3C2A | 2.3±0.2 | 2 | 1 |
| BA24 | PILRB | 3.0±0.0 | 2 | 1 |
| insula | PILRB | 5.1±0.8 | 2 | 1 |
| BA46 | PINK1 | 2.7±0.3 | 3 | 1 |
| BA46 | PINK1-AS | 2.7±0.3 | 3 | 1 |
| BA24 | PJA2 | 3.0±0.6 | 4 | 1 |
| postputamen | PJA2 | 2.7±0.5 | 5 | 1 |
| cerebellum | PKP4 | 2.4±0.4 | 3 | 1 |
| BA24 | PLBD2 | 2.5±0.0 | 2 | 1 |
| raphaenucleus | PLCB1 | 2.9±0.4 | 2 | 1 |
| cerebellum | PLK2 | 4.6±0.9 | 2 | 1 |
| BA10 | POLR2B | 4.0±1.4 | 3 | 1 |
| insula | POLR3F | 3.8±1.2 | 2 | 1 |
| BA24 | POMP | 4.6±1.3 | 2 | 1 |
| BA24 | PPFIBP1 | 4.2±1.2 | 2 | 1 |
| hippocampus | PPHLN1 | 3.5±1.0 | 2 | 1 |
| BA10 | PPP1CB | 2.4±0.3 | 2 | 1 |
| amygdala | PPP1R11 | 3.0±0.5 | 2 | 1 |
| insula | PPP1R11 | 2.3±0.1 | 2 | 1 |
| BA22 | PPP1R12B | 2.5±0.1 | 2 | 1 |
| raphaenucleus | PPP3CA | 4.6±2.2 | 4 | 1 |
| BA10 | PPT1 | 2.8±0.1 | 2 | 1 |
| BA10 | PRDM2 | 2.7±0.5 | 2 | 1 |
| insula | PRDM2 | 3.3±0.8 | 2 | 1 |
| insula | PREX1 | 2.2±0.2 | 4 | 1 |
| amygdala | PRICKLE2 | 3.2±0.4 | 2 | 1 |
| BA24 | PRKACB | 2.9±0.7 | 6 | 1 |
| BA24 | PRKCB | 3.9±2.0 | 6 | 1 |
| BA10 | PRPF8 | 3.2±1.1 | 5 | 1 |
| BA24 | PRPF8 | 5.2±0.9 | 2 | 1 |
| BA10 | PRRC2C | 3.5±1.1 | 6 | 1 |
| hippocampus | PRRC2C | 4.6±0.6 | 2 | 1 |
| BA10 | PSIP1 | 2.9±0.4 | 3 | 1 |
| postputamen | PTTG1IP | 2.9±0.1 | 2 | 1 |
| postputamen | PYGB | 4.5±1.7 | 2 | 1 |
| raphaenucleus | QDPR | 2.6±0.4 | 3 | 1 |
| postputamen | QKI | 3.5±0.6 | 3 | 1 |
| BA10 | RAB21 | 5.7±0.4 | 2 | 1 |
| hippocampus | RAB2A | 2.4±0.2 | 3 | 1 |
| postputamen | RAB3C | 2.1±0.0 | 2 | 1 |
| postputamen | RAC1 | 2.3±0.1 | 2 | 1 |
| raphaenucleus | RALGAPB | 5.8±2.5 | 2 | 1 |
| BA10 | RANBP2 | 4.3±1.5 | 2 | 1 |
| hippocampus | RBFOX1 | 2.5±2.1 | 110 | 1 |
| raphaenucleus | RBM26 | 5.6±1.5 | 2 | 1 |
| BA46 | RCAN2 | 4.1±1.6 | 5 | 1 |
| BA24 | RDH11 | 3.7±0.2 | 2 | 1 |
| BA24 | RERE | 2.4±0.3 | 3 | 1 |
| raphaenucleus | REV3L | 2.2±0.0 | 2 | 1 |
| BA22 | RHCE | 2.4±0.0 | 2 | 1 |
| hippocampus | RIMKLA | 3.9±0.7 | 3 | 1 |
| raphaenucleus | RIMS2 | 4.8±0.1 | 2 | 1 |
| cerebellum | RNF207 | 3.1±0.2 | 2 | 1 |
| BA10 | RNF216 | 2.5±0.0 | 2 | 1 |
| insula | RNF39 | 2.3±0.1 | 2 | 1 |
| BA24 | RP11-1012A1.4 | 3.7±0.2 | 2 | 1 |
| amygdala | RP11-129B22.1 | 3.2±0.4 | 2 | 1 |
| postputamen | RP11-131L23.1 | 2.3±0.1 | 3 | 1 |
| raphaenucleus | RP11-154D6.1 | 3.7±0.9 | 3 | 1 |
| BA10 | RP11-159D12.5 | 4.6±1.4 | 2 | 1 |
| BA24 | RP11-269G24.3 | 4.2±0.8 | 2 | 1 |
| postputamen | RP11-283I3.6 | 2.4±0.2 | 2 | 1 |
| amygdala | RP11-318M2.2 | 2.3±0.2 | 3 | 1 |
| amygdala | RP11-318M2.3 | 2.4±0.1 | 2 | 1 |
| BA10 | RP11-386G11.3 | 2.3±0.0 | 2 | 1 |
| raphaenucleus | RP11-440L14.1 | 2.5±0.4 | 3 | 1 |
| raphaenucleus | RP11-463C8.4 | 3.8±2.4 | 8 | 1 |
| BA10 | RP11-463J10.3 | 2.9±0.7 | 3 | 1 |
| postputamen | RP11-473C19.1 | 2.7±0.4 | 2 | 1 |
| postputamen | RP11-490D19.6 | 2.1±0.1 | 2 | 1 |
| postputamen | RP11-509E10.1 | 2.8±0.4 | 7 | 1 |
| hippocampus | RP11-565P22.6 | 4.2±1.6 | 3 | 1 |
| amygdala | RP11-589P10.7 | 2.3±0.1 | 2 | 1 |
| BA10 | RP11-598P20.5 | 3.3±0.6 | 2 | 1 |
| hippocampus | RP11-619F23.2 | 3.2±0.4 | 2 | 1 |
| BA24 | RP11-69L16.4 | 2.6±0.0 | 2 | 1 |
| BA10 | RP11-6O2.4 | 2.9±0.6 | 3 | 1 |
| BA10 | RP11-731J8.2 | 4.1±1.0 | 2 | 1 |
| amygdala | RP11-746M1.1 | 4.0±0.1 | 2 | 1 |
| hippocampus | RP11-785H5.1 | 6.5±1.8 | 2 | 1 |
| hippocampus | RP11-785H5.2 | 6.5±1.8 | 2 | 1 |
| BA24 | RP11-862L9.3 | 3.8±1.3 | 4 | 1 |
| cerebellum | RP11-862L9.3 | 6.8±2.6 | 2 | 1 |
| raphaenucleus | RP11-981G7.1 | 4.4±1.4 | 2 | 1 |
| raphaenucleus | RP13-225O21.2 | 3.5±0.6 | 3 | 1 |
| raphaenucleus | RP3-331H24.5 | 3.7±0.9 | 3 | 1 |
| insula | RPL21P3 | 3.8±1.2 | 2 | 1 |
| BA22 | RPS20 | 3.2±0.7 | 3 | 1 |
| raphaenucleus | RPS20 | 3.1±0.3 | 3 | 1 |
| BA10 | RPS23 | 2.6±0.1 | 2 | 1 |
| amygdala | RPS6KA2 | 3.4±1.1 | 4 | 1 |
| hippocampus | SBF2 | 3.0±0.6 | 2 | 1 |
| hippocampus | SBF2-AS1 | 3.0±0.6 | 2 | 1 |
| cerebellum | SCLT1 | 2.5±0.3 | 2 | 1 |
| postputamen | SCLT1 | 3.2±0.8 | 2 | 1 |
| hippocampus | SCN1A | 4.2±1.8 | 4 | 1 |
| hippocampus | SCN3A | 3.4±0.1 | 2 | 1 |
| amygdala | SCN4B | 2.3±0.2 | 2 | 1 |
| BA10 | SCN4B | 2.5±0.1 | 2 | 1 |
| postputamen | SCN4B | 3.2±0.5 | 2 | 1 |
| BA22 | SCP2 | 2.7±0.3 | 2 | 1 |
| BA22 | SENP2 | 3.6±0.2 | 3 | 1 |
| hippocampus | SENP2 | 3.8±1.2 | 2 | 1 |
| cerebellum | SEPT3 | 5.0±1.0 | 2 | 1 |
| raphaenucleus | SEPT3 | 4.7±1.8 | 2 | 1 |
| BA46 | SERINC1 | 3.3±0.5 | 2 | 1 |
| postputamen | SETD6 | 3.6±1.4 | 4 | 1 |
| BA10 | SETX | 4.8±1.9 | 6 | 1 |
| postputamen | SH3BP1 | 5.0±0.4 | 2 | 1 |
| hippocampus | SHISA6 | 2.8±0.1 | 2 | 1 |
| hippocampus | SLAIN1 | 2.9±0.6 | 2 | 1 |
| postputamen | SLC25A27 | 2.7±0.5 | 3 | 1 |
| BA10 | SLC25A36 | 3.5±0.5 | 2 | 1 |
| cerebellum | SLC2A12 | 2.8±0.4 | 2 | 1 |
| hippocampus | SLC44A1 | 3.9±1.9 | 5 | 1 |
| BA22 | SLC6A1 | 2.5±0.1 | 2 | 1 |
| BA46 | SLC8A1 | 5.8±1.0 | 2 | 1 |
| BA24 | SLC8A3 | 2.7±0.0 | 2 | 1 |
| BA10 | SMAD3 | 3.8±1.0 | 3 | 1 |
| hippocampus | SMU1 | 3.5±0.6 | 3 | 1 |
| hippocampus | SNAP91 | 3.9±0.5 | 2 | 1 |
| amygdala | SNHG14 | 5.8±2.5 | 9 | 1 |
| postputamen | SNHG14 | 5.3±2.4 | 7 | 1 |
| BA10 | SNHG16 | 4.1±0.2 | 2 | 1 |
| cerebellum | SNRNP200 | 2.5±0.0 | 2 | 1 |
| postputamen | SNX27 | 3.1±0.9 | 3 | 1 |
| raphaenucleus | SOGA1 | 5.1±2.2 | 6 | 1 |
| hippocampus | SORT1 | 2.2±0.1 | 2 | 1 |
| postputamen | SOS1 | 2.8±0.1 | 2 | 1 |
| hippocampus | SOWAHA | 2.8±0.0 | 2 | 1 |
| BA24 | SPARC | 2.4±0.3 | 2 | 1 |
| insula | SPARC | 2.7±0.2 | 2 | 1 |
| hippocampus | SPG20 | 3.4±0.5 | 2 | 1 |
| BA22 | SPHKAP | 2.3±0.1 | 2 | 1 |
| BA24 | SPHKAP | 4.0±1.4 | 2 | 1 |
| BA24 | SPIRE1 | 3.3±0.4 | 2 | 1 |
| BA22 | SPOCK2 | 2.3±0.1 | 2 | 1 |
| BA24 | SPOCK2 | 3.1±1.1 | 4 | 1 |
| postputamen | SPP1 | 4.7±1.8 | 3 | 1 |
| BA46 | SPTBN1 | 3.6±0.6 | 2 | 1 |
| BA24 | SSR1 | 2.6±0.0 | 2 | 1 |
| BA10 | ST6GALNAC2 | 4.1±0.2 | 2 | 1 |
| raphaenucleus | ST8SIA1 | 2.7±0.0 | 2 | 1 |
| hippocampus | STARD13 | 4.4±0.1 | 2 | 1 |
| amygdala | STON2 | 5.6±1.6 | 2 | 1 |
| hippocampus | STX16 | 2.8±0.0 | 2 | 1 |
| hippocampus | STX16-NPEPL1 | 2.8±0.0 | 2 | 1 |
| BA10 | SUMF1 | 2.8±0.9 | 6 | 1 |
| postputamen | SV2B | 3.8±0.0 | 2 | 1 |
| BA24 | SYNJ1 | 5.6±0.2 | 2 | 1 |
| insula | SYNJ1 | 2.4±0.1 | 2 | 1 |
| BA10 | SYNM | 3.5±1.4 | 4 | 1 |
| postputamen | SYT11 | 2.6±0.5 | 3 | 1 |
| BA10 | TAF2 | 5.4±1.0 | 2 | 1 |
| BA24 | TANC2 | 4.2±0.8 | 2 | 1 |
| BA24 | TAOK1 | 4.6±2.3 | 4 | 1 |
| BA10 | TCHP | 3.8±2.0 | 6 | 1 |
| raphaenucleus | TCHP | 3.4±1.4 | 5 | 1 |
| hippocampus | TCP11L1 | 2.3±0.1 | 2 | 1 |
| BA10 | TESPA1 | 2.8±0.3 | 2 | 1 |
| raphaenucleus | TF | 2.5±0.2 | 2 | 1 |
| BA10 | TMBIM6 | 3.1±0.4 | 4 | 1 |
| postputamen | TMEM246 | 2.1±0.1 | 2 | 1 |
| BA22 | TMEM50A | 2.4±0.0 | 2 | 1 |
| postputamen | TNIK | 2.5±0.4 | 2 | 1 |
| raphaenucleus | TNPO2 | 3.6±0.6 | 2 | 1 |
| BA10 | TNS3 | 5.0±2.1 | 2 | 1 |
| insula | TPM1 | 3.0±0.4 | 2 | 1 |
| amygdala | TRPS1 | 2.1±0.1 | 3 | 1 |
| hippocampus | TSPYL4 | 2.2±0.1 | 2 | 1 |
| BA10 | TTL | 3.4±1.1 | 4 | 1 |
| insula | TTTY14 | 6.4±2.8 | 2 | 1 |
| BA22 | TXN2 | 2.2±0.0 | 2 | 1 |
| BA24 | TXN2 | 5.5±0.3 | 2 | 1 |
| raphaenucleus | TXNL1 | 2.6±0.0 | 2 | 1 |
| BA24 | UBE2L3 | 2.8±0.6 | 3 | 1 |
| amygdala | UBE3A | 5.1±2.5 | 4 | 1 |
| BA10 | UBE3A | 5.7±3.2 | 4 | 1 |
| cerebellum | UBR3 | 4.7±1.7 | 2 | 1 |
| BA10 | UFM1 | 5.6±2.2 | 2 | 1 |
| hippocampus | UGT8 | 3.3±0.4 | 2 | 1 |
| hippocampus | UHMK1 | 3.5±1.0 | 3 | 1 |
| cerebellum | UQCRB | 2.4±0.2 | 3 | 1 |
| raphaenucleus | USP24 | 4.6±0.6 | 4 | 1 |
| hippocampus | USP47 | 4.7±1.4 | 2 | 1 |
| postputamen | USP47 | 2.1±0.0 | 2 | 1 |
| raphaenucleus | WAC | 2.8±0.3 | 2 | 1 |
| BA10 | WASF1 | 2.3±0.1 | 3 | 1 |
| amygdala | WDFY3 | 3.5±0.7 | 2 | 1 |
| insula | WDR41 | 3.2±0.8 | 3 | 1 |
| BA10 | XRCC5 | 4.7±1.5 | 3 | 1 |
| hippocampus | YLPM1 | 2.6±0.2 | 3 | 1 |
| BA24 | YWHAB | 4.8±1.7 | 2 | 1 |
| postputamen | YWHAB | 3.1±0.6 | 2 | 1 |
| cerebellum | YWHAG | 3.4±0.9 | 2 | 1 |
| postputamen | YWHAG | 4.5±0.1 | 2 | 1 |
| BA10 | ZDHHC21 | 3.5±0.9 | 2 | 1 |
| BA10 | ZEB1 | 5.5±0.8 | 2 | 1 |
| postputamen | ZEB1 | 4.7±0.5 | 2 | 1 |
| amygdala | ZFYVE20 | 2.3±0.1 | 2 | 1 |
| cerebellum | ZFYVE26 | 2.2±0.1 | 3 | 1 |
| amygdala | ZIM2 | 4.1±1.5 | 3 | 1 |
| BA24 | ZNF106 | 3.0±0.4 | 2 | 1 |
| hippocampus | ZNF326 | 2.7±0.0 | 2 | 1 |
| postputamen | ZNF385D | 2.6±0.3 | 2 | 1 |
| BA22 | ZNF391 | 4.6±0.3 | 2 | 1 |
| amygdala | ZNF462 | 2.6±0.2 | 2 | 1 |
| hippocampus | ZNF626 | 2.3±0.1 | 2 | 1 |
| BA10 | ZNF638 | 3.3±1.1 | 3 | 1 |
| cerebellum | ZNF638 | 2.1±0.1 | 3 | 1 |
| raphaenucleus | ZNF641 | 2.8±0.1 | 2 | 1 |
| BA10 | ZNF697 | 4.2±0.3 | 3 | 1 |
| cerebellum | ZNF83 | 2.5±0.3 | 3 | 1 |
| BA22 | ZNF91 | 7.1±2.2 | 2 | 1 |
| raphaenucleus | ZNF91 | 3.8±1.6 | 7 | 1 |
| BA10 | ZYG11B | 3.0±0.2 | 2 | 1 |
