# Supplementary figures and images for: RNA sequencing of transcriptomes in human brain regions: protein-coding and non-coding RNAs, isoforms and alleles
Source: BMC Genomics. 2015 Nov 23;16:990. doi: 10.1186/s12864-015-2207-8 (PMC4657279; doi:10.1186/s12864-015-2207-8)

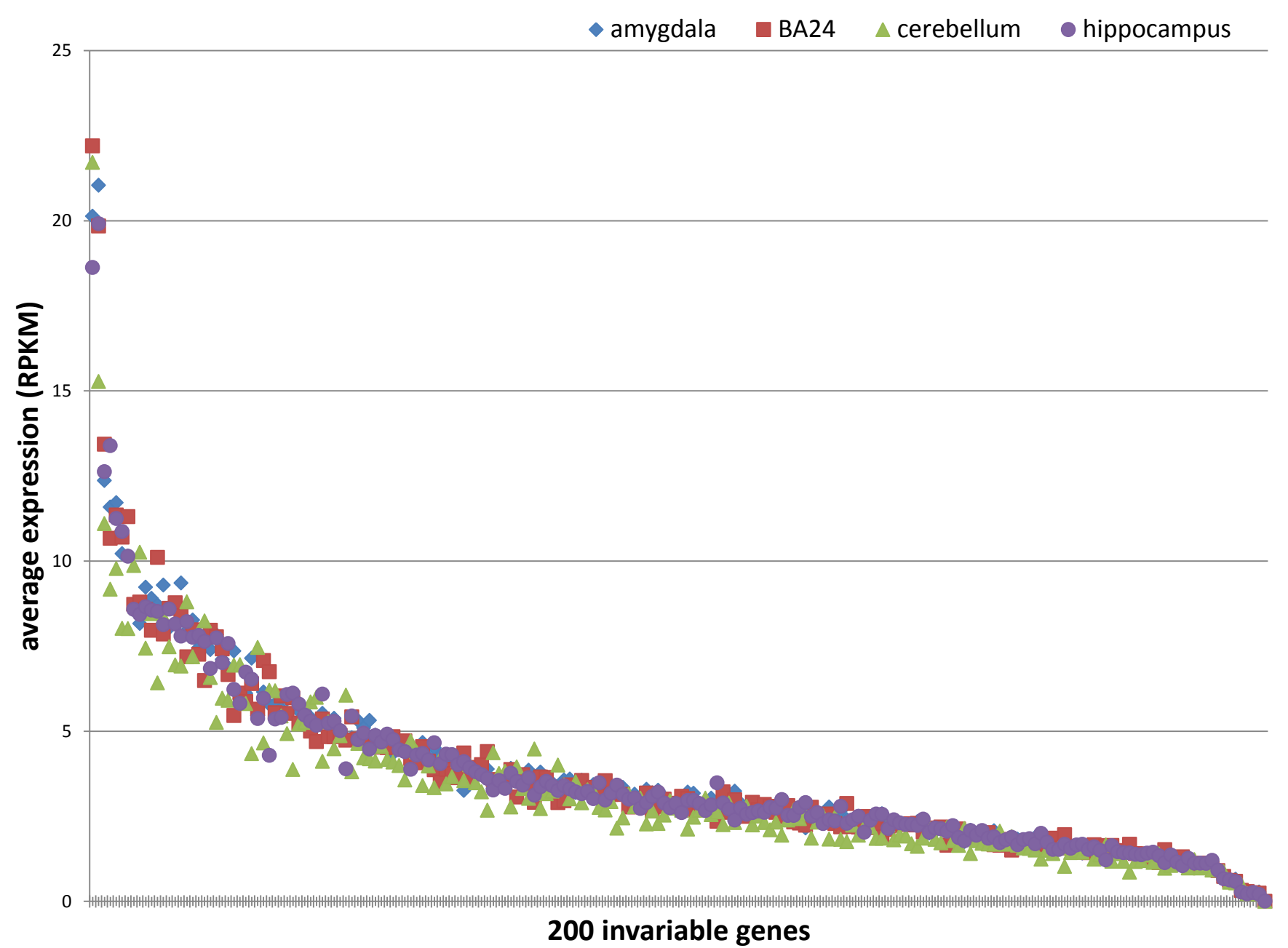

Supplement: Additional file 2: Figure S1 — Average expression of invariable genes in 4 GTEx brains. Shows the variation of 200 invariable genes expressed in 4 GTEx brain regions. (PDF 189 kb) [file 12864_2015_2207_MOESM2_ESM.pdf]

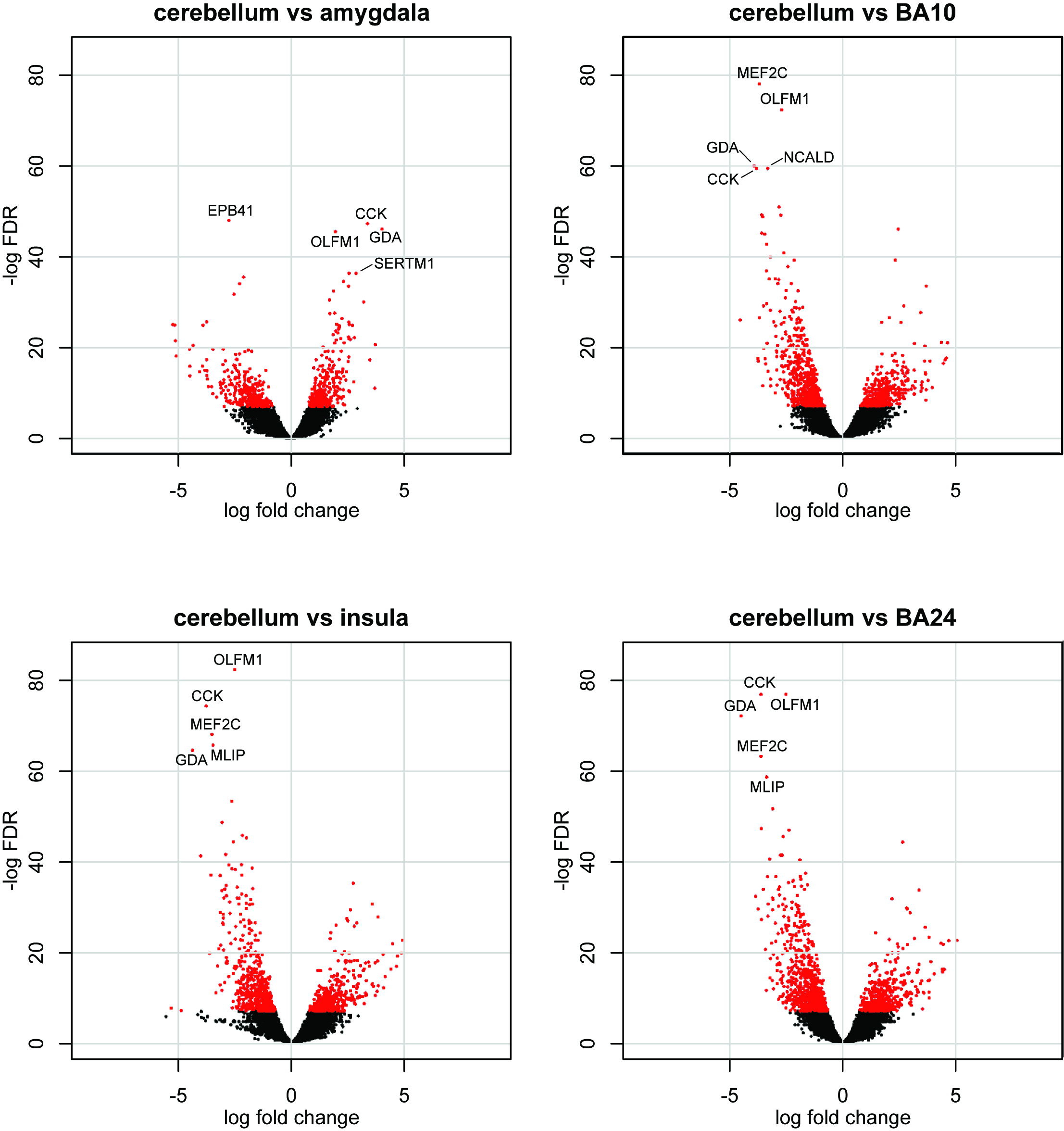

Supplement: Additional file 3: Figure S2 — Visualization of the results of differential expression analysis for four pairs of brain regions with highest differences in gene expression. log fold-change (x-axis) and corresponding –log FDR (y-axis) are shown for each gene. Genes differentially expressed (FDR < 0.05) are marked in red and top five DE genes for each pair are indicated. (TIF 915 kb) [file 12864_2015_2207_MOESM3_ESM.tif]

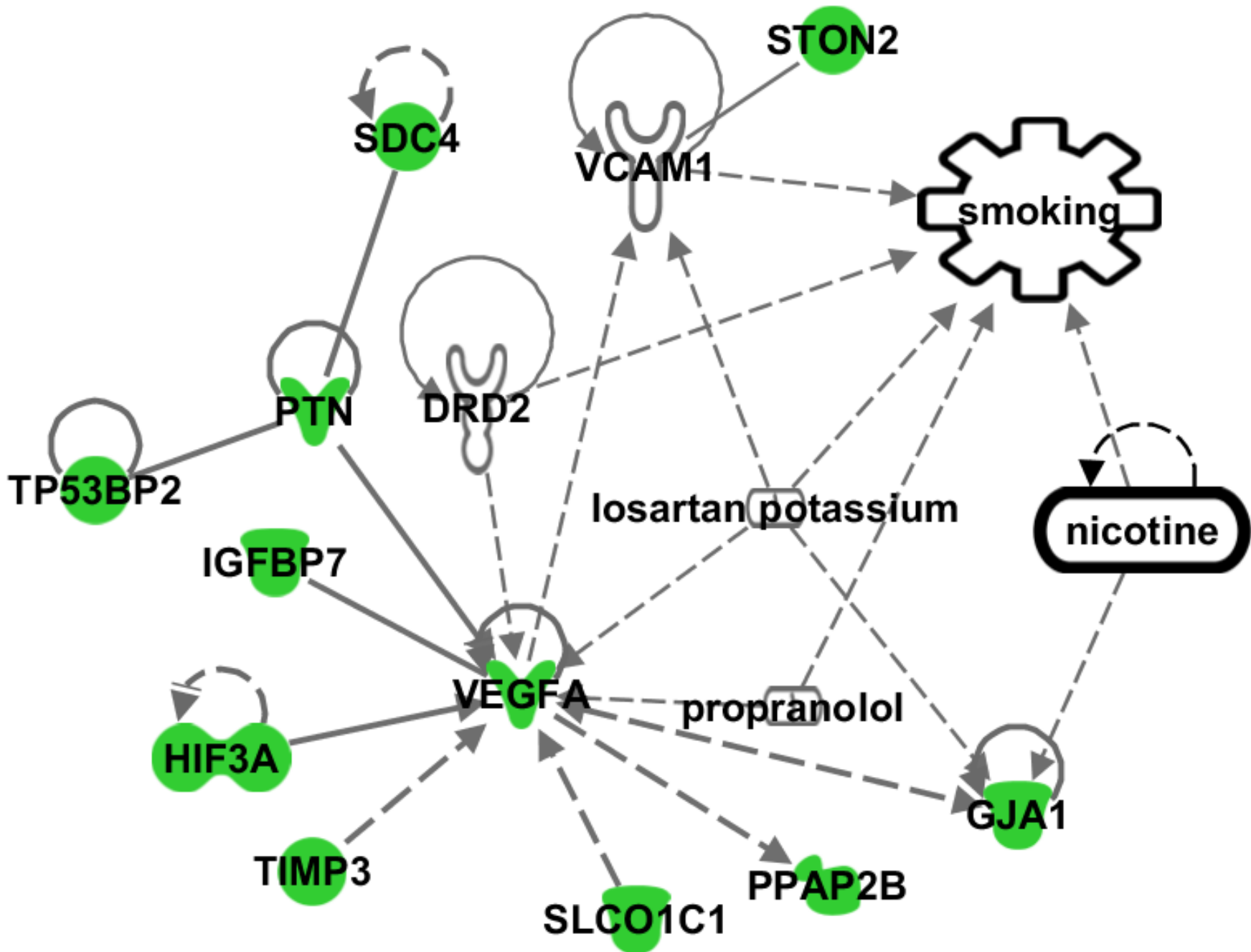

Supplement: Additional file 4: Figure S3 — Pathway relating VEGF and other differentially expressed genes to smoking. Displays a pathway generated by Ingenuity Pathway Analysis using genes differentially expressed between smokers and nonsmokers in BA46 that connect to smoking and smoking related molecules. Differentially expressed genes are highlighted in green and smoking related molecules are outlined in purple. (PDF 80 kb) [file 12864_2015_2207_MOESM4_ESM.pdf]

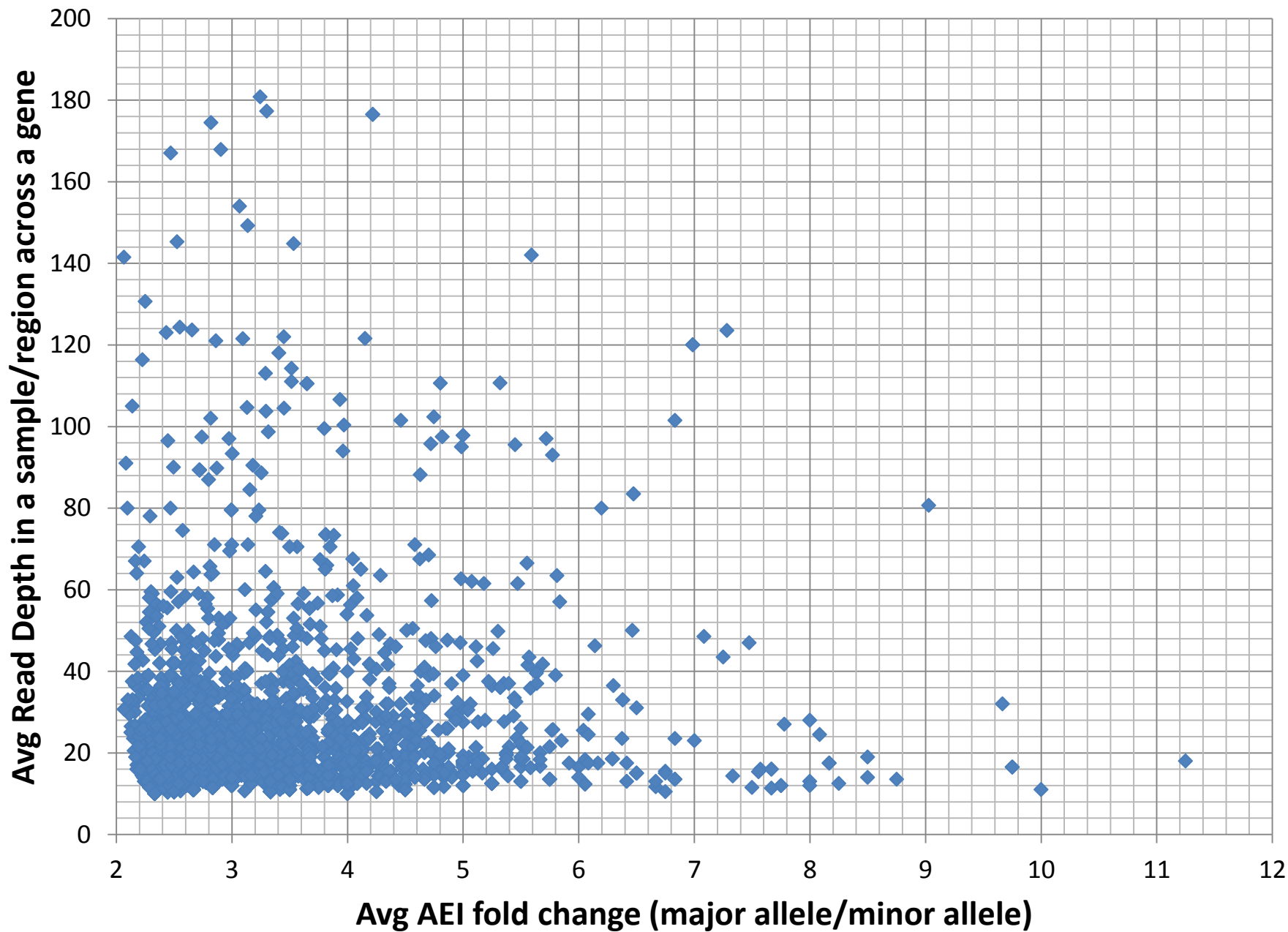

Supplement: Additional file 5: Figure S4 — Plot of AEI ratio versus read depth across SNP. Displays the average magnitude of the allelic expression fold-change of a gene for a particular sample/region compared to the average read depth for SNPs in the gene. SNPs with low coverage tend to have more extreme AEI. (PDF 211 kb) [file 12864_2015_2207_MOESM5_ESM.pdf]

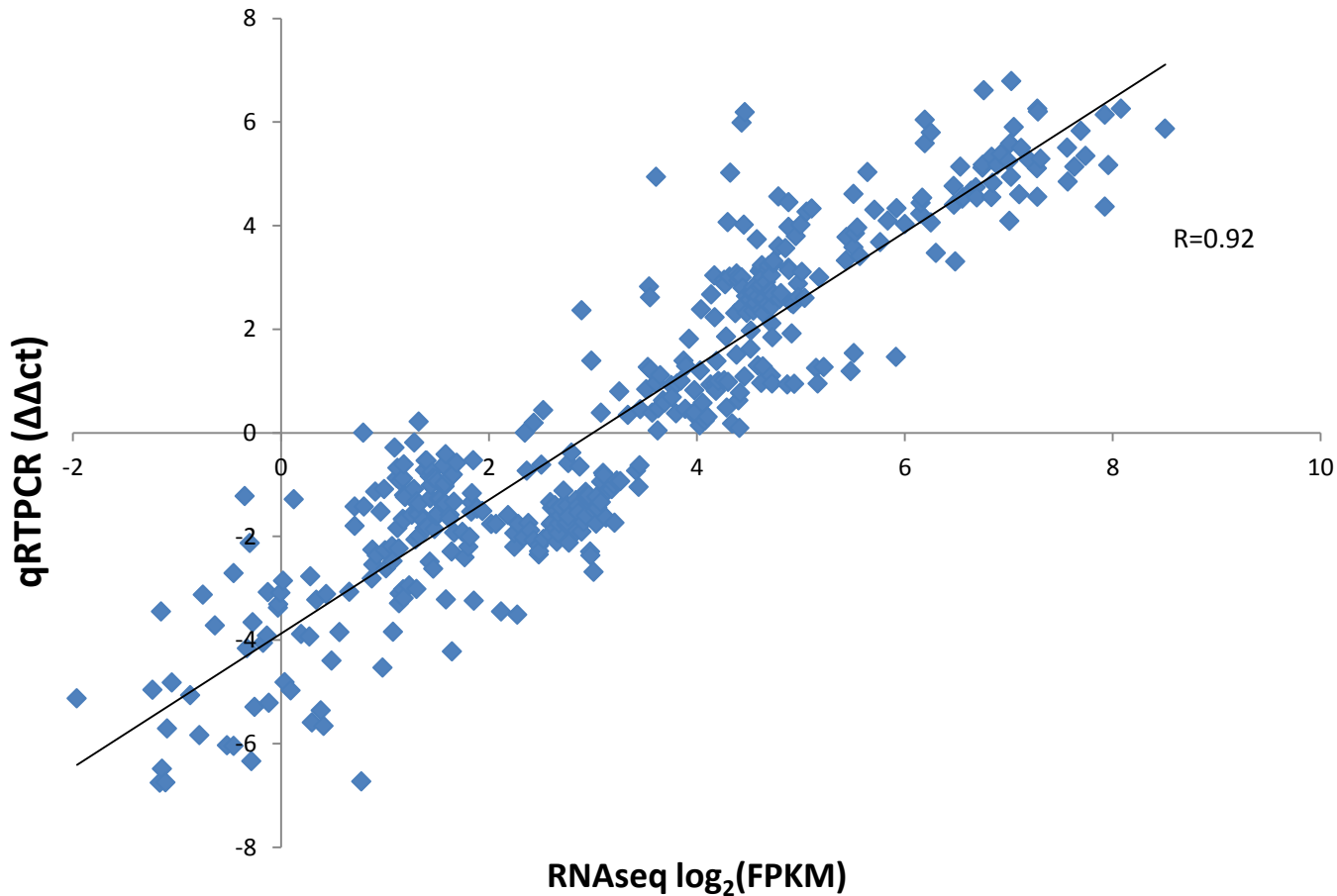

Supplement: Additional file 6: Figure S5 — Confirmation of expression levels by qRTPCR. qRTPCR was performed to correlate the FPKM of 3 invariably expressed (AGO1, SPEN, SRSF11) and 3 nicotine candidate genes (HIF3A, SLC1A3, NRXN3) for nearly all 100 samples. qRTPCR was quantified using the ∆Δct method and using the average of the three invariable genes for normalization. Across brain regions, we can correlate the RTPCR measured expression with the RNAseq measured expression. After a log transformation, the overall Pearson correlation is 0.92. (PDF 173 kb) [file 12864_2015_2207_MOESM6_ESM.pdf]

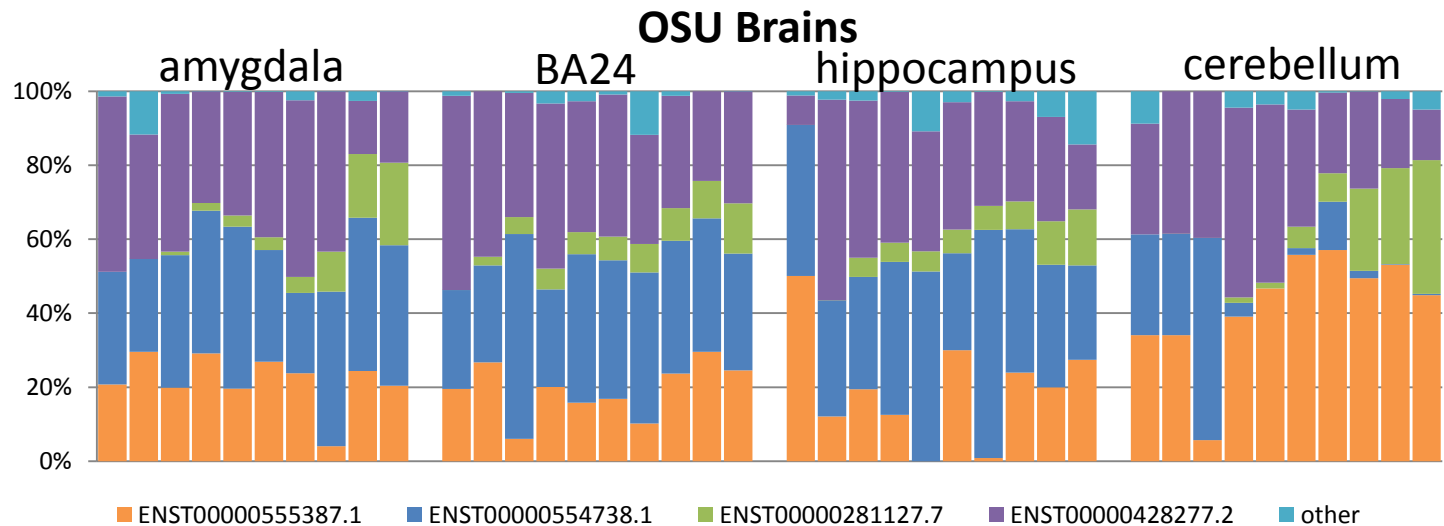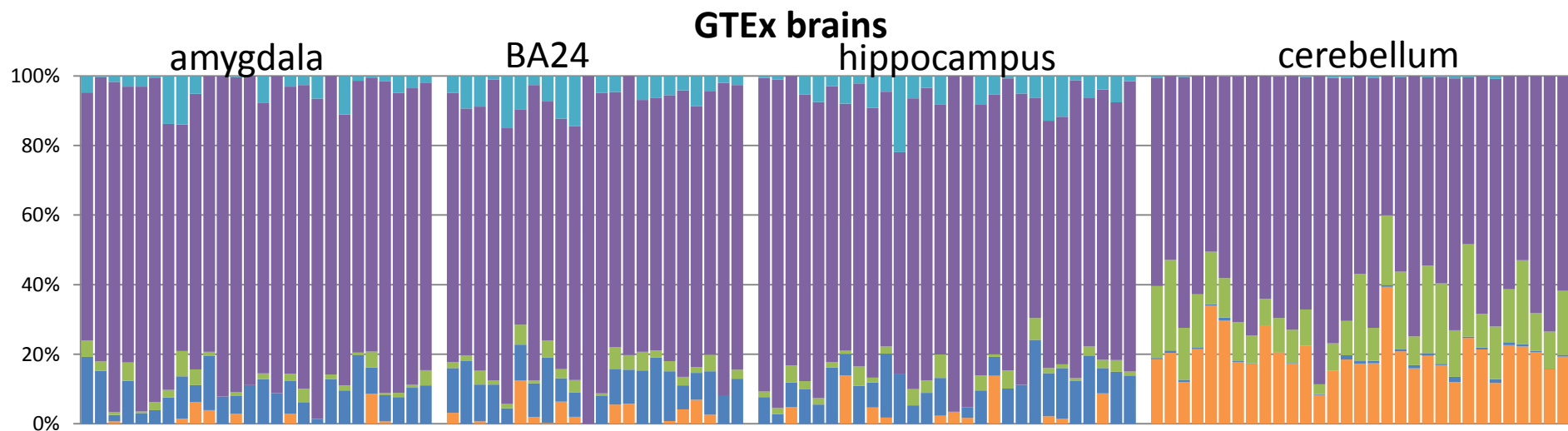

Supplement: Additional file 7: Figure S6 — Comparison of NRXN3 isoform representation between OSU and GTEx brains. The ratio of the blue and orange isoforms favors ENST00000554738.1 in both GTEx and OSU in the cerebellum and favors ENST00000555387.1 in the other 3 overlapping regions. ENST00000554738.1 corresponds to NRXN3_dup5, and ENST00000555387.1 corresponds to NRXN3_dup0. The protein coding version ENST00000428277.2 is highly represented in GTEx brains likely due to poly-A selection. (PDF 176 kb) [file 12864_2015_2207_MOESM7_ESM.pdf]
